# Overcoming primary non-response to infliximab in ulcerative colitis: monocyte-derived PTGS2 as a therapeutic target

**DOI:** 10.3389/fimmu.2026.1957588

**Published:** 2026-10-20

**Authors:** Dan Pu, Xiaojuan Kang, Lingzhu Gou, Xiaoli Li, Pengfei Wang, Guoqing Qi, Jun-Li Liu, Dekui Zhang

**Affiliations:** 1 Key Laboratory of Digestive Diseases, The Second Hospital and Clinical Medical School, Lanzhou University, Lanzhou, Gansu, China; 2 Department of Gastroenterology, The Second Hospital and Clinical Medical School, Lanzhou University, Lanzhou, Gansu, China; 3 MeDiC Program, The Research Institute of McGill University Health Centre and Division of Endocrinology and Metabolism, Department of Medicine, McGill University, Montreal, QC, Canada

**Keywords:** infliximab, monocytes, neutrophil chemotaxis, primary non-response, PTGS2, ulcerative colitis

## Abstract

**Background:**

Primary non-response (PNR) to infliximab (IFX) remains a major therapeutic challenge in ulcerative colitis (UC). However, the molecular mechanisms underlying this treatment failure remains unelucidated. Therefore, there is an urgent need to identify the key drivers of PNR and develop effective interventions to improve therapeutic outcomes.

**Methods:**

Human colonic RNA-sequencing data were analyzed to identify molecular features associated with PNR to IFX. Immune infiltration analysis, clinical sample validation, virtual gene knockout, and single-cell RNA sequencing were performed to characterize the immune landscape, identify the key regulatory molecule, and elucidate its underlying mechanisms and cellular source. Network pharmacology and molecular docking were employed to identify a potential therapeutic strategy targeting the key regulatory molecule, followed by mechanistic and functional validation in cell-based assays and therapeutic evaluation in a DSS-induced murine colitis model.

**Results:**

Excessive colonic neutrophil infiltration marked PNR in UC prior to IFX treatment. PTGS2 positively correlated with neutrophil infiltration and was predominantly expressed in colonic CD14^+^S100A9^+^ inflammatory monocytes. Virtual knockout of PTGS2 altered genes related to neutrophil chemotaxis, which were primarily enriched in the NF-κB signaling axis. Gegen Qinlian Decoction (GQD), a traditional Chinese herbal formula, was identified to target PTGS2 through multiple bioactive constituents, with quercetin exhibiting the highest binding affinity. Quercetin suppressed LPS-induced PTGS2 expression in inflammatory monocytes, thereby inhibiting the NF-κB/CXCL8 signaling and attenuating neutrophil chemotaxis, as further supported by PTGS2 knockdown and overexpression experiments. *In vivo*, GQD markedly reduced PTGS2 expression and neutrophil accumulation in the inflamed colon, thereby significantly ameliorating disease severity and enhancing IFX responsiveness in DSS-induced colitis.

**Conclusion:**

Monocyte-derived PTGS2 contributes to primary IFX non-response by promoting neutrophil recruitment in UC. GQD improves IFX responsiveness by suppressing PTGS2-associated inflammatory signaling and neutrophil recruitment, highlighting its potential as a therapeutic strategy to enhance IFX efficacy.

## 1 Introduction

Ulcerative colitis (UC), a principal clinical subtype of inflammatory bowel disease (IBD), is characterized by chronic, relapsing inflammation of the colonic and rectal mucosa (1). The incidence and prevalence of UC have increased worldwide in recent decades, posing a growing burden on healthcare systems and patients (2, 3).

Biologic therapy is the first-line treatment for moderate-to-severe UC, acting by selectively targeting key inflammatory mediators involved in disease pathogenesis (4). In recent years, an increasing number of novel biologic agents, including vedolizumab (5), ustekinumab (6), risankizumab (7), and mirikizumab (8), have been introduced into the clinical management of UC. Nevertheless, owing to its well-established efficacy, broad accessibility, and relatively low cost, infliximab (IFX) remains one of the most widely used biologic therapies for moderate-to-severe UC worldwide (9). IFX is a chimeric human-mouse monoclonal antibody that specifically binds to both soluble and transmembrane tumor necrosis factor-α (TNF-α), thereby neutralizing its biological activity (10). However, clinical studies have shown that approximately 30% of patients exhibit non-response to IFX (11), substantially limiting its overall therapeutic benefit in patients with UC.

IFX treatment failure can be classified into primary non-response (PNR) and secondary loss of response (LOR). PNR is defined as the failure to achieve a clinical response during the induction phase of treatment (12), whereas LOR refers to the gradual decline in therapeutic response after an initial successful induction (13). Considerable studies have elucidated the mechanisms underlying LOR to IFX, with pharmacokinetic failure and immunogenicity recognized as the two major contributors (14–16). In contrast, current studies on PNR have largely focused on identifying immune-associated features and predictive biomarkers (17–20), while the molecular mechanisms driving PNR remain poorly understood. Moreover, effective strategies to overcome PNR are still lacking. Therefore, there is an unmet clinical need to identify the key molecular drivers of IFX primary non-response, and to develop effective therapeutic strategies for the improvement of treatment responsiveness in UC.

In this study, we elucidated the molecular landscape and immune microenvironment features associated with PNR to IFX in UC, and revealed the essential role of inflammatory monocyte-derived PTGS2 in the pathogenesis of PNR. We further identified Gegen Qinlian Decoction (GQD), a traditional Chinese herbal formula containing multiple bioactive constituents that collectively target PTGS2, as a promising adjunctive therapeutic strategy for enhancing IFX responsiveness. These findings were validated both *in vitro* and *in vivo* experiments, providing mechanistic insights into the therapeutic efficacy of GQD in overcoming PNR to IFX.

## 2 Materials and methods

### 2.1 Patients and samples

IFX-treated patients with moderate-to-severe UC from the Second Hospital & Clinical Medical School of Lanzhou University were retrospectively enrolled. The study protocol was approved by the Medical Ethics Committee from the Second Hospital & Clinical Medical School of Lanzhou University (Approval No. 2026A-980). All included patients received IFX as their first biologic therapy. All patients (n=44) received a standard dose of 5 mg/kg of IFX and underwent clinical assessment at weeks 0 and 14 of treatment. Disease activity was evaluated using the Modified Mayo Disease Activity Index (MMDAI) (21). Treatment response was assessed based on changes in MMDAI and Mayo Endoscopic Score (MES). Specifically, patients achieving a reduction of at least 3 points and 30% from baseline MMDAI scores, together with a decrease of ≥1 point in the MES (22) were classified as primary responders to IFX (n=23). In contrast, those failing to meet these response criteria were classified as the primary non-responder group (n=21). Baseline demographic and clinical characteristics, including age, sex, disease duration, disease extent according to the Montreal classification (E1, ulcerative proctitis; E2, left-sided colitis; and E3, extensive colitis), baseline MMDAI, baseline MES, and IFX induction dose, were collected from the medical records and compared between primary responders and non-responders (**Supplementary Table 1**).

### 2.2 Animals care

Animal experiment in this study was approved by the Animal Ethics Committee from the Second Hospital & Clinical Medical School of Lanzhou University (Lanzhou, China) (Approval No. D2026-507). It was conducted with the NIH Guide for the Care and Use of Laboratory Animals. Male C57BL/6J mice (8–10 weeks of age, weighing 20 ± 2 g) were obtained from the Lanzhou Veterinary Research Institute, Chinese Academy of Agricultural Sciences (Lanzhou, China). Mice were maintained in a specific pathogen-free facility under environmental conditions (22 ± 2 °C, 50 ± 5% relative humidity, 12 h light/dark cycle) with ad libitum access to standard food and water.

### 2.3 Animal grouping and treatment

Mice were randomly assigned to five experimental groups (n = 5 per group): wild-type control (WT), DSS-induced colitis (DSS), GQD monotherapy (DSS+GQD), anti-TNF-α monotherapy (DSS+IFX), and combination therapy (DSS+IFX+GQD). The sample size was determined based on preliminary experiments, with consideration of the Reduction principle of the 3Rs for animal research. Treatment administration was performed by one investigator according to group allocation, while outcome assessments were conducted by other members of the research team according to predefined criteria and standardized procedures.

Specifically, experimental colitis was induced by administering 2.5% dextran sulfate sodium (DSS; 36–50 kDa, 60316ES76, Yeasen, China) in mice’s drinking water for 8 consecutive days, after which the DSS solution was replaced with normal drinking water. As IFX is directed against human TNF-α and is not suitable for neutralizing murine TNF-α *in vivo*, a mouse-specific *in vivo* anti-TNF-α antibody (T258, Thermo Fisher Scientific) was used as a surrogate for IFX treatment in mice. Based on preliminary dose-finding experiments, mice received intraperitoneal injections of anti-TNF-α antibody (2.5 mg/kg) on days 3, 5, and 7. This dose did not significantly ameliorate DSS-induced colitis when administered alone and was therefore selected to establish an experimental setting of limited anti-TNF-α therapeutic efficacy for subsequent evaluation of the effect of GQD on anti-TNF-α treatment. GQD was administered by oral gavage at a dose of 3.86 g/kg/day from day 3 to day 9. During the experimental period, body weight, stool consistency, and rectal bleeding were recorded daily. The severity of colitis was evaluated using the Disease Activity Index (DAI) according to previously published criteria (23). All mice were euthanized on day 10, and colonic samples were collected for subsequent analyses. The method of euthanasia for the mice was as follows: mice were deeply anesthetized with sodium pentobarbital (60mg/kg, intraperitoneally). After loss of pedal and corneal reflexes, cervical dislocation was performed for euthanasia. Death was confirmed by respiratory and cardiac arrest.

### 2.4 Preparation of Gegen Qinlian decoction

Gegen Qinlian Decoction (GQD) was prepared according to previously established protocols (24, 25). Briefly, GQD was prepared using standardized dispensing granules purchased from EFONG Pharmaceutical Co., Ltd. (Foshan, Guangdong, China). These dispensing granules are standardized herbal extracts manufactured under controlled extraction procedures with a fixed extract-to-crude-herb ratio to ensure batch-to-batch consistency. The granules corresponding to *Radix Puerariae*, *Scutellariae Radix*, *Coptidis Rhizoma*, and *Liquorice* were mixed according to the traditional crude-herb ratio of 5:3:3:2 (w/w/w/w). Detailed quality control information for the four dispensing granules, including batch numbers, granule specification, and corresponding crude-herb equivalents, is provided in **Supplementary Table 2**. For *in vivo* treatment, the prepared GQD was dissolved in distilled water and administered orally at a dose of 3.86 g/kg/day.

### 2.5 Immunohistochemistry assay

Immunohistochemical staining was performed on formalin-fixed paraffin-embedded human or mouse colonic tissue sections (5 μm). Following deparaffinization, rehydration, antigen retrieval, and serum blocking, sections were incubated overnight at 4 °C with primary antibodies against MPO (1:500; YT5351, Immunoway) or PTGS2 (1:1000, ab179800, Abcam). After incubation with HRP-conjugated secondary antibodies, immunoreactivity was visualized using a DAB detection kit (abs9210, Absin). Stained slides were digitized using a KFBIO Digital Pathology Slide Scanner (KF-PRO-005-EX). Quantitative analysis was performed using the IHC Analyzer plugin in ImageJ as previously described (26). Three representative fields were randomly selected from each section. Staining intensity was classified as high positive, positive, low positive, or negative. The final immunohistochemical score was calculated using the formula: IHC score = (high positive×3) + (positive×2) + (low positive×1), with the mean value from the three fields used for statistical analysis.

### 2.6 H&E staining and histological assessment

Mouse colonic specimens were excised, fixed in 4% paraformaldehyde, and processed for paraffin embedding. Serial sections (5 μm) were prepared and stained with hematoxylin and eosin (H&E; G1120, Solarbio, China) to assess the colonic mucosal structure and inflammatory injury. Histological severity was quantified according to the Histologic Activity Index (HAI) described previously (27). It incorporates scores for inflammatory infiltration, goblet cell depletion, crypt abscess formation, and epithelial erosion. Each parameter was graded from 0 to 3, and the total score was calculated by summing individual component scores. All slides were evaluated in a blinded fashion by two independent pathologists.

### 2.7 Cell culture and treatment

Human THP-1 monocytic cells and HL-60 cells were obtained from the National Collection of Authenticated Cell Cultures (NCACC, Shanghai, China). THP-1 cells were maintained in RPMI-1640 medium supplemented with 10% fetal bovine serum (FBS), whereas HL-60 cells were maintained in IMDM supplemented with 20% FBS. Both cell lines were cultured at 37°C in a humidified incubator containing 5% CO_2_.

For stimulation experiments, THP-1 cells were pretreated with quercetin (25 μM; T2174, TargetMol, China) for 1 h, followed by stimulation with lipopolysaccharide (LPS; 1.5 μg/mL; L4391, Sigma-Aldrich, USA) for 24 h in the continued presence or absence of quercetin. As the quercetin stock solution was prepared in DMSO, the corresponding control groups received an equal volume of DMSO as the vehicle control. The concentration of quercetin used in subsequent experiments was determined based on cell viability assessed by the CCK-8 assay.

For PTGS2 loss-of-function experiments, PTGS2-specific small interfering RNA (siPTGS2) and the corresponding negative-control siRNA (si-NC) were obtained from Tsingke Biotechnology (China). THP-1 cells were transfected with siPTGS2 or si-NC using TSnanofect RNAi-Specific Transfection Reagent (DLH101; Tsingke Biotechnology, China). At 24 h after transfection, cells were stimulated with LPS (1.5 μg/mL) for an additional 24 h. For PTGS2 overexpression and rescue experiments, the PTGS2 overexpression plasmid (OE-PTGS2) and the corresponding empty vector were obtained from Tsingke Biotechnology (China). THP-1 cells were transfected with OE-PTGS2 or the empty vector using Lipofectamine LTX Reagent with PLUS Reagent (15338100; Invitrogen, USA). After 24 h after transfection, cells were treated with LPS and/or quercetin as indicated. Following treatment, cells were harvested for subsequent analyses. PTGS2 knockdown and overexpression efficiencies were confirmed by Western blotting.

### 2.8 Western blot assay

Western blot analysis was performed to assess protein expression in THP-1 cells and colonic tissues. Total cellular proteins were extracted from THP-1 cells or homogenized colonic tissues using ice-cold RIPA lysis buffer supplemented with protease and phosphatase inhibitors, and protein concentrations were determined using a BCA Protein Assay Kit (BL521, Biosharp, China). Equal amounts of protein were separated by SDS-PAGE and subsequently transferred onto polyvinylidene difluoride membranes. After blocking with 5% bovine serum albumin (BSA; CW0054S, CoWin, China) for 1 h at room temperature, membranes were incubated overnight at 4 °C with primary antibodies against PTGS2 (1:1000, ab179800, Abcam), NF-κB p65 (1:1000, ab32536, Abcam), phospho-NF-κB p65 (Ser536) (1:1000, ab76302, Abcam), CXCL8 (1:1000, ab289967, Abcam), and GAPDH (1:20,000, 10494-1-AP, Proteintech). Following incubation with HRP-conjugated secondary antibodies, protein bands were detected using an enhanced chemiluminescence kit (BL520A, Biosharp, China) and visualized using a chemiluminescence imaging system.

### 2.9 Cell migration and chemotaxis assay

Neutrophil cell migration and chemotaxis was evaluated using a Transwell-based system. For the quercetin treatment experiments, THP-1 monocytes were seeded into the lower chambers of 24-well Transwell plates and stimulated with LPS (1.5 μg/mL; L4391, Sigma-Aldrich, USA) in the presence or absence of quercetin (25 μM; T2174, TargetMol, China) for 24 h. An equal volume of DMSO was added to the corresponding control groups as the vehicle control. For the PTGS2 loss-of-function experiments, THP-1 cells transfected with PTGS2 siRNA or negative control siRNA were stimulated with LPS (1.5 μg/mL) for 24 h before the chemotaxis assay. For the PTGS2 overexpression and rescue experiments, THP-1 cells transfected with OE-PTGS2 or empty vector were stimulated with LPS (1.5 μg/mL) in the presence or absence of quercetin (25 μM) for 24 h before the chemotaxis assay. In the upper chamber, HL-60 cells were differentiated into neutrophil-like cells by treatment with 1.3% dimethyl sulfoxide (DMSO) for 5 days, then harvested and resuspended in RPMI 1640 medium before being seeded into the upper chambers of Transwell inserts with 5.0μm polycarbonate membranes (Cat. 3421, Corning, USA). The cells were co-cultured for an additional 20 h at 37 °C in a humidified incubator containing 5% CO_2_. Non-migrated cells remaining on the upper surface of the membrane were gently removed with a cotton swab. Cells that had migrated to the lower surface of the membrane were fixed with 4% paraformaldehyde, stained with 0.1% crystal violet, and photographed under a light microscope. Migrated cells were quantified by counting three randomly selected microscopic fields for each insert.

### 2.10 Cell viability assay

The effect of quercetin on THP-1 cell viability was evaluated using a Cell Counting Kit-8 (CCK-8) assay. THP-1 cells were seeded in 96-well plates and treated with quercetin at concentrations of 0, 2.5, 5, 10, 15, 20, 25, 50 and 100 μM for 25 h. Subsequently, 10 μL of CCK-8 reagent was added to each well, followed by incubation at 37°C for 2 h. Absorbance was measured at 450 nm using a microplate reader, and cell viability was calculated relative to the untreated control.

### 2.11 Transcriptomic dataset integration and differential expression analysis

Three publicly available transcriptomic datasets (GSE12251, GSE14580, GSE73661) were retrieved from the Gene Expression Omnibus (GEO) database (https://www.ncbi.nlm.nih.gov/geo/) for the evaluation of IFX responsiveness. Baseline colonic biopsy samples from patients with moderate-to-severe UC were collected before IFX treatment (week 0). In the original clinical studies, the included patients had clinically comparable baseline disease activity. Patients were categorized as responders or non-responders according to their clinical and endoscopic responses assessed within 14 weeks of treatment initiation. In total, 70 baseline colonic samples were included, comprising 28 responders and 42 non-responders from GSE12251 (12 vs 11), GSE14580 (8 vs 16), and GSE73661 (8 vs 15). Following the batch-effect correction by limma and sva packages in R, the three datasets were normalized and merged. Differential expression analysis between responders and non-responders was subsequently performed using the limma package. Genes with an adjusted *P* value < 0.05 and an absolute log2 fold change (|log2FC|) > 1 were defined as differentially expressed genes (DEGs). The expression signature of DEGs were visualized using heatmaps generated with the pheatmap package, while the top 30 DEGs ranked by fold change were further displayed in a bubble plot.

### 2.12 Gene ontology enrichment analysis

Gene Ontology (GO) enrichment analysis was performed on the identified DEGs using the clusterProfiler and DOSE packages in R. Enriched GO terms were categorized into three domains, including biological process (BP), cellular component (CC), and molecular function (MF). Terms with an adjusted *P* value < 0.05 were considered significantly enriched, and were visualized as bar plots using the ggplot2 package.

### 2.13 KEGG-based gene set enrichment analysis

To explore characteristic biological pathways associated with IFX responsiveness, GSEA was performed using KEGG gene sets with the clusterProfiler package in R. Pathways with an FDR-adjusted *P* value < 0.05 were considered significantly enriched. Enrichment plots were generated using the enrichplot and ggplot2 packages to visualize the normalized enrichment score (NES) and enrichment profiles of representative pathways.

### 2.14 Immune cell infiltration and correlation analyses

Immune cell composition in colonic tissues was estimated using the CIBERSORT algorithm with 1000 permutations and quantile normalization. Wilcoxon rank-sum test was used to compare the relative abundance of immune cell subsets between responders and non-responders. Samples with a *P* value < 0.05 were retained for subsequent analyses, resulting in 68 of the 70 samples being included in the final immune infiltration analysis (26 responders and 42 non-responders). Immune cell populations were visualized as bar plots in R. Associations between the top 10 DEGs and neutrophils were assessed using Spearman’s rank correlation test in R. Correlation coefficients and corresponding *P* values were calculated for each gene-neutrophil pair. Significant correlations (*P* < 0.05) were visualized as scatter plots with fitted regression lines and marginal density distributions using the ggplot2, ggpubr, and ggExtra packages. Next, spearman correlation coefficients and corresponding *P* values between PTGS2 expression and each immune cell subset were calculated and visualized as lollipop plots in R.

### 2.15 Single-cell transcriptomic data processing and cell clustering

Single-cell RNA sequencing data from inflamed and uninflamed colonic mucosa of patients with UC were obtained from the GEO database (GSE231993), comprising four inflamed and four uninflamed colonic tissue samples. The Seurat package was used to process raw count matrices in R. Gene expression data were normalized using LogNormalize, and the top 1500 highly variable genes were identified for downstream analysis. Principal component analysis (PCA) was then performed for dimensionality reduction. Harmony algorithm was employed to correct batch effects among samples. Cell clustering was conducted and visualized by t-distributed stochastic neighbor embedding (t-SNE).

### 2.16 Single-cell annotation, cellular composition and gene expression analysis

Using the SingleR package, cell-type annotation was performed based on publicly available reference datasets, including Human Primary Cell Atlas, Blueprint/ENCODE, Monaco Immune Data, Novershtern Hematopoietic Data, Database Immune Cell Expression Data, and other hematopoietic reference datasets. Cluster-specific marker genes were identified using the FindAllMarkers function in Seurat. Differentially expressed genes were DEGs were defined by |log2FC| > 1 and adjusted *P* < 0.05. Cell distributions across inflamed and uninflamed tissues were visualized using t-SNE plots. The distribution of PTGS2, CD14, and S100A9 among annotated cell populations was examined using the Seurat package. Gene expression patterns were visualized by t-SNE feature plots. Bubble plots were generated to compare the average expression levels and percentages of expressing cells across different cell clusters.

### 2.17 Virtual knockout analysis

The PTGS2 virtual knockout analysis was conducted using the scTenifoldKnk package based on the processed scRNA-seq data from inflamed colonic tissues in UC. Raw count matrix was extracted from the Seurat object, and the top 5000 highly variable genes together with PTGS2 were included in the analysis. The scTenifoldKnk workflow was performed with 10 sub-networks and 500 randomly sampled cells per network. Differentially regulated genes after PTGS2 virtual knockout were identified using an adjusted *P* value < 0.05 and showed through a volcano graph The top 20 genes ranked by fold change were visualized as bar plots, while the proportion of significantly regulated genes among background genes was displayed using a pie chart.

### 2.18 Functional enrichment analysis of PTGS2-regulated genes

Genes significantly altered following PTGS2 virtual knockout were intersected with genes associated with IFX non-response by the use of VennDiagram package in R. Next, GO enrichment analysis was performed on these overlapping genes by the clusterProfiler package. Significantly enriched GO terms were identified according to *P* value and adjusted *P* value thresholds, and were visualized using tree plots generated with the enrichplot package. KEGG pathway enrichment analysis was also performed using the clusterProfiler package, and enriched pathways were displayed as bubble plots.

### 2.19 Network pharmacology analysis of GQD

Network pharmacology analysis was performed to identify the bioactive compounds of GQD and their potential therapeutic targets. The active constituents of *Radix Puerariae* (Gegen), *Scutellariae Radix* (Huangqin), *Coptidis Rhizoma* (Huanglian), and *Licorice* (Gancao) were retrieved from the Traditional Chinese Medicine Systems Pharmacology Database (TCMSP, https://www.tcmsp-e.com/). Compounds with oral bioavailability (OB) ≥ 30% and drug-likeness (DL) ≥ 0.18 were considered bioactive ingredients. Subsequently, potential target genes of these compounds were obtained from the TCMSP database. Using the VennDiagram package in R, these identified target genes were then intersected with DEGs associated with IFX non-response to identify candidate therapeutic targets of GQD.

### 2.20 PPI network and compound-target interaction analysis

Protein-protein interaction (PPI) data for the overlapping genes discovered in 2.18 section were retrieved from the STRING database (https://cn.string-db.org/). Genes were ranked based on their connectivity. Subsequently, Cytoscape software was used to construct and visualize the compound-target interaction network, which illustrated the relationships between bioactive compounds of GQD and the overlapping target genes, as well as the herbal origin of each compound. The degree of each node in the compound-target interaction network was calculated according to the number of interacting nodes.

### 2.21 Molecular docking analysis

Molecular docking analysis was performed to evaluate the binding affinity between PTGS2 and the six bioactive compounds (Quercetin, Stigmasterol, Kaempferol, Isorhamnetin, Oroxylin A and Wogonin) with the highest degree values identified from the compound-target interaction network. The two-dimensional (2D) structures of these compounds were retrieved from the PubChem database (https://pubchem.ncbi.nlm.nih.gov/) and converted into optimized three-dimensional (3D) conformations using Chem3D software. The crystal structure of PTGS2 (PDB ID: 5KIR; resolution: 2.70 Å), which contains the co-crystallized selective COX-2 inhibitor rofecoxib, was obtained from the Protein Data Bank (PDB, http://www.rcsb.org/). Protein preparation was performed using PyMOL software by removing water molecules and co-crystallized ligands. Hydrogen atoms were subsequently added to the receptor using AutoDockTools (MGLTools), and the prepared receptor and ligands were converted into PDBQT format. The docking search space was centered at x = 31.054, y = 24.187, and z = 23.582 Å. Finally, Molecular docking was performed using AutoDock Vina. The energy range was set to 5 kcal/mol, and a maximum of 20 binding modes was generated for each ligand. The docking pose with the lowest binding free energy was selected as the optimal binding conformation for each compound-PTGS2 complex.

### 2.22 Statistical analysis

Bioinformatic analyses were performed using R software (version 4.3.2), and experimental data were analyzed using GraphPad Prism (version 9.0). Data are presented as mean ± SEM. Data normality and homogeneity of variance were assessed before statistical comparisons. For normally distributed data with equal variances, comparisons between two groups were performed using an unpaired Student’s t-test, while multiple-group comparisons were analyzed by one-way ANOVA followed by Tukey’s *post hoc* test. For non-normally distributed data, comparisons between two groups were performed using the Mann-Whitney U test, while multiple-group comparisons were analyzed using the Kruskal-Wallis test followed by Dunn’s multiple-comparison test. Two-factor experiments were analyzed by two-way ANOVA followed by Bonferroni’s multiple-comparison test. For baseline clinical characteristics, continuous variables are presented as median (IQR) and were compared using the Mann-Whitney U test, while categorical variables are presented as n (%) and were compared using Pearson’s χ² test. Correlations were assessed using Spearman’s rank correlation analysis. A two-sided P < 0.05 was considered statistically significant. Statistical significance was defined as *P < 0.05, **P < 0.01, ***P < 0.001, and ****P < 0.0001.

## 3 Results

### 3.1 Baseline colonic immune cell recruitment is enhanced in primary non-responders to IFX

To investigate baseline molecular differences between IFX responders and non-responders, three colonic biopsy microarray datasets (GSE12251, GSE14580, and GSE73661) from patients with moderate-to-severe UC were integrated into a single cohort following batch-effect correction (**Figure 1A**). Differential expression analysis revealed that, despite presenting with a similar degree of disease activity prior to IFX treatment, patients who failed to respond to IFX displayed a distinct baseline colonic transcriptional profile compared with responders. The heatmap highlights the top 10 differentially expressed genes (DEGs), including *IL13RA2*, *TNFRSF11B*, *STC1*, *IL11*, *TFPI2*, *WNT5A*, *ACSL4*, *HSD11B1*, *PTGS2*, and *INHBA* (**Figure 1B**; **Supplementary Table 3**).

**Figure 1:**
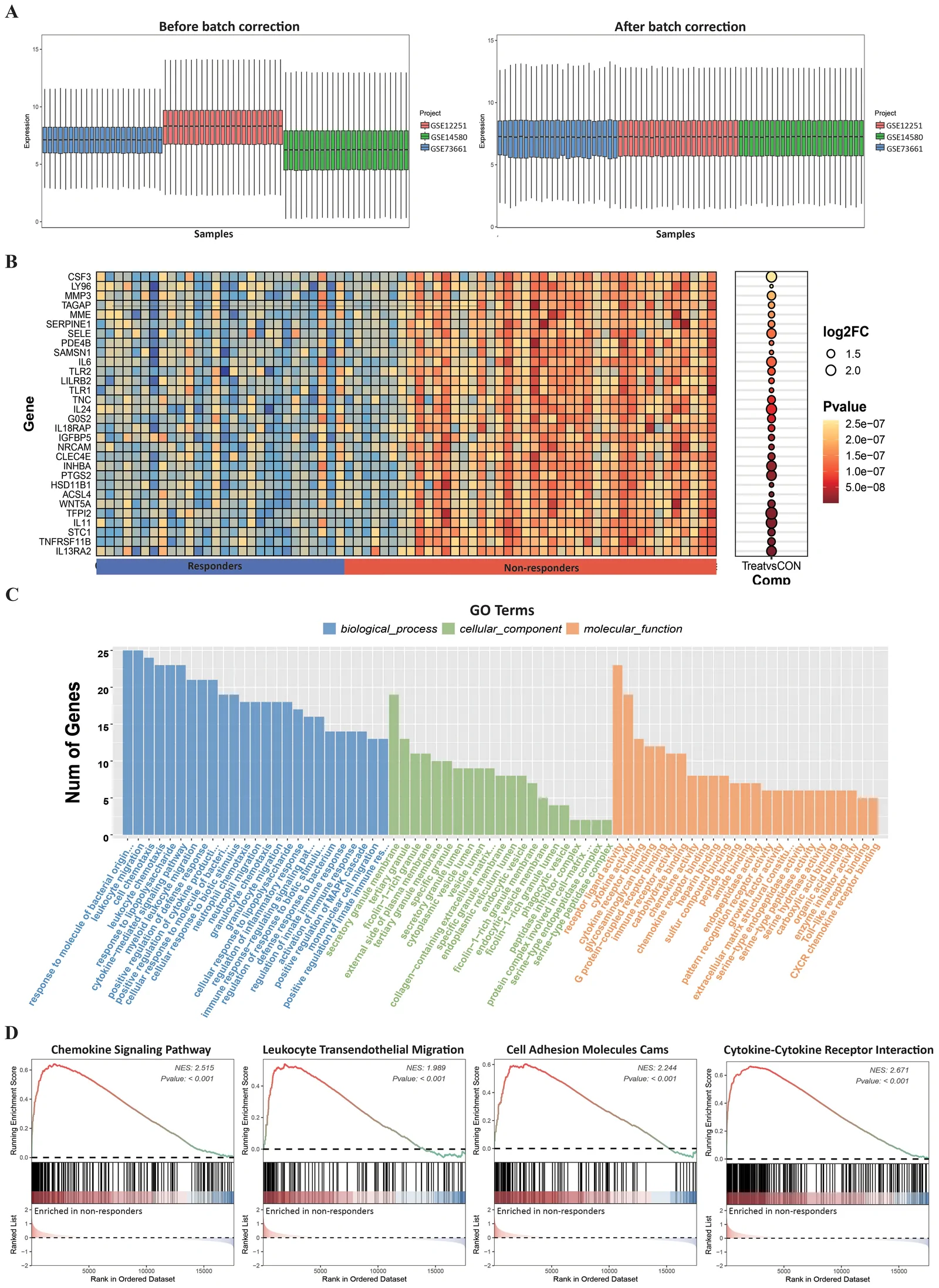
Baseline colonic immune features associated with primary IFX non-response in UC. **(A)** Boxplots of gene expression distributions across the three datasets before and after batch correction. **(B)** Heatmap of DEGs in baseline colonic tissues between IFX responders and non-responders. **(C)** Bar plot of the top enriched GO terms among DEGs. **(D)** GSEA plots of the top enriched KEGG pathways in baseline colonic tissues from IFX non-responders.

Gene Ontology (GO) enrichment analysis further characterized the biological functions associated with the DEGs (**Figure 1C**). In the Biological Process (BP) category, DEGs were predominantly enriched in leukocyte chemotaxis and migration, particularly in neutrophils. In the Cellular Component (CC) category, significant enrichment was observed in inflammation-related structures involved in neutrophil recruitment and transendothelial migration, including tertiary granules, ficolin-1-rich granules, and the external side of the plasma membrane. The enriched Molecular Function (MF) terms were primarily associated with receptor-ligand interactions implicated in immune cell trafficking, including immune receptor activity, chemokine activity, and chemokine receptor binding. Consistent with these findings, KEGG-based Gene Set Enrichment Analysis (GSEA) further demonstrated significant enrichment of multiple immune cell chemotaxis-related pathways in colonic tissues from IFX non-responders, including Chemokine Signaling Pathway, Leukocyte Transendothelial Migration, Cell Adhesion Molecules, and Cytokine-Cytokine Receptor Interaction (**Figure 1D**). These findings indicate that enhanced colonic immune cell chemotaxis, particularly neutrophil recruitment, represents a prominent molecular feature of primary IFX non-response and is already established before treatment initiation, independent of baseline disease activity.

### 3.2 Predominant colonic neutrophil infiltration marks primary IFX non-response and correlates with PTGS2 expression

The immune infiltration landscape was then characterized using colonic transcriptomic data from patients with UC to identify immune cell populations associated with primary IFX non-response. Neutrophils, mast cells, dendritic cells, M1 macrophages, and a subset of naïve CD4^+^ T cells were enriched in the colonic mucosa of IFX non-responders compared with responders (**Figure 2A**). Among these immune cell populations, neutrophils exhibited the most pronounced increase in infiltration. This observation was consistent with the GO enrichment analysis, which identified neutrophil chemotaxis and neutrophil migration as the most significantly enriched biological processes associated with IFX non-response (**Figure 1C**). In addition, MPO immunohistochemical staining also confirmed the markedly increased neutrophil infiltration in baseline colonic tissues from IFX non-responders compared with responders (**Figure 2B**).

**Figure 2:**
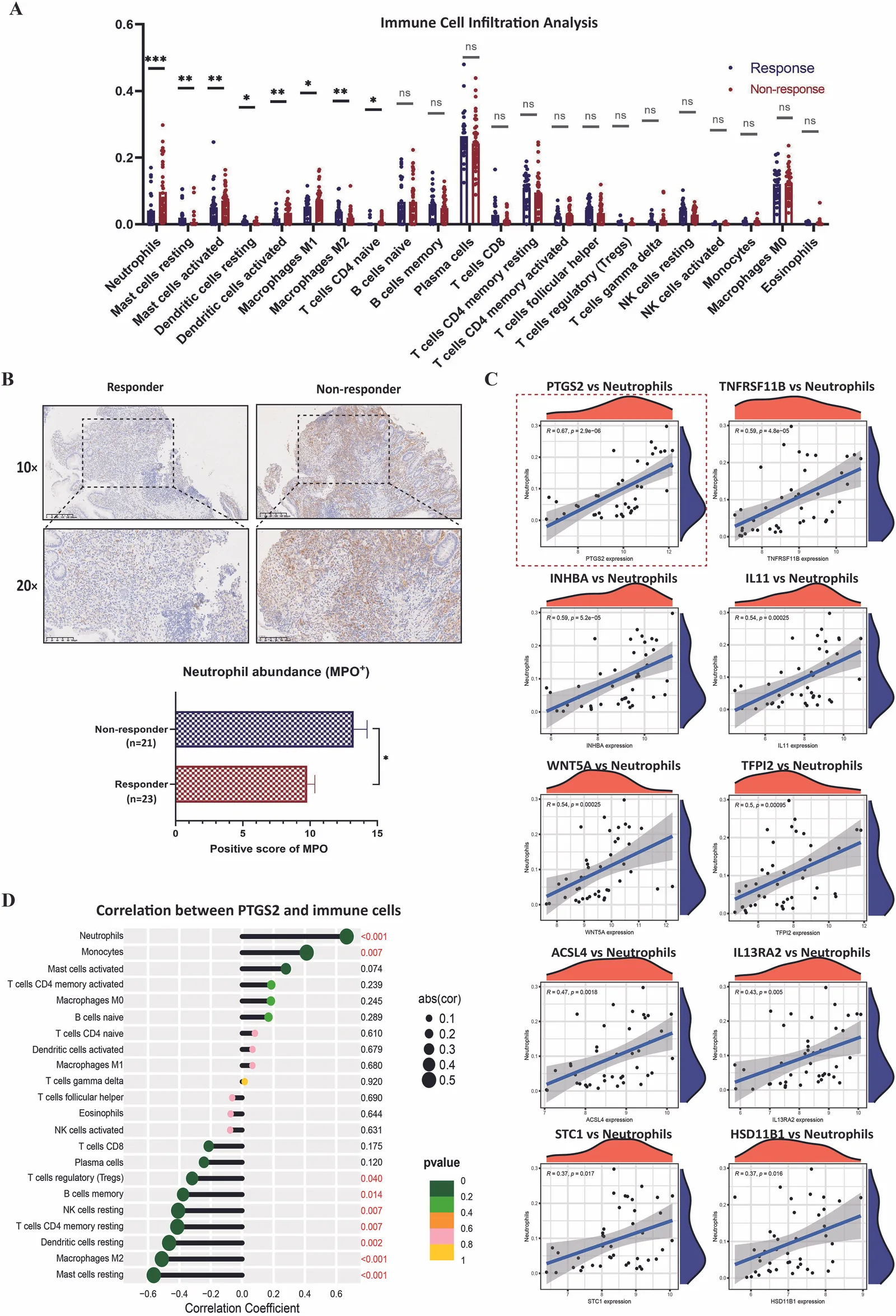
Immune cell infiltration and its correlations with IFX non-response-related genes in UC. **(A)** Bar plot of immune cell infiltration in baseline colonic tissues from IFX responders and non-responders. **(B)** Representative immunohistochemical images and quantitative analysis of MPO-positive neutrophils in baseline colonic tissues from IFX responders and non-responders. **(C)** Correlation analysis between neutrophil infiltration and the top 10 DEGs associated with IFX non-response. **(D)** Correlation analysis between *PTGS2* and the abundance of infiltrating immune cells in the inflamed colon. Data are presented as mean ± SEM. Following assessment of data normality, two-group comparisons were performed using a Mann-Whitney U test. Correlations were assessed using Spearman’s rank correlation analysis. A two-sided P < 0.05 was considered statistically significant. Statistical significance was defined as *P < 0.05, **P < 0.01, and ***P < 0.001.

As neutrophils exhibited the most pronounced increase in IFX non-responders, we next explored potential molecular determinants associated with neutrophil recruitment. Correlation analysis between neutrophil infiltration and the top 10 DEGs identified PTGS2 as the gene most strongly and positively associated with neutrophil infiltration, exhibiting both the highest correlation coefficient and the most significant *P* value (**Figure 2C**). Moreover, among all immune cell subsets associated with *PTGS2* expression, neutrophils showed the strongest positive correlation, followed by monocytes (**Figure 2D**). In contrast, resting mast cells, M2 macrophages, and resting dendritic cells showed significant negative correlations with *PTGS2* expression. Therefore, increased colonic neutrophil infiltration emerged as a characteristic feature of PNR to IFX, and its strong association with *PTGS2* suggested that *PTGS2* may contribute to IFX non-response by promoting neutrophil recruitment.

### 3.3 Colonic virtual knockout of PTGS2 affects neutrophil chemotaxis and NF-κB signaling

To characterize the role of PTGS2 in neutrophil recruitment, we performed a virtual PTGS2 knockout analysis using whole-colon transcriptomic data. A total of 179 genes were significantly affected following *PTGS2* deletion, accounting for approximately 3.6% of all genes included in the analysis (**Figures 3A, B**; **Supplementary Table 4**). Among the most prominently altered genes were *TYROBP*, *FCER1G*, *CD68*, *AIF1*, *HLA-DRA*, *HLA-DRB1*, *HLA-DPA1*, *HLA-DPB1*, and complement-related genes (*C1QA*, *C1QB* and *C1QC*) (**Figure 3C**). Notably, these genes are classically associated with myeloid cell activation, innate immune responses, and antigen presentation, indicating a potential regulatory role of *PTGS2* in shaping myeloid inflammatory programs within the colonic microenvironment.

**Figure 3:**
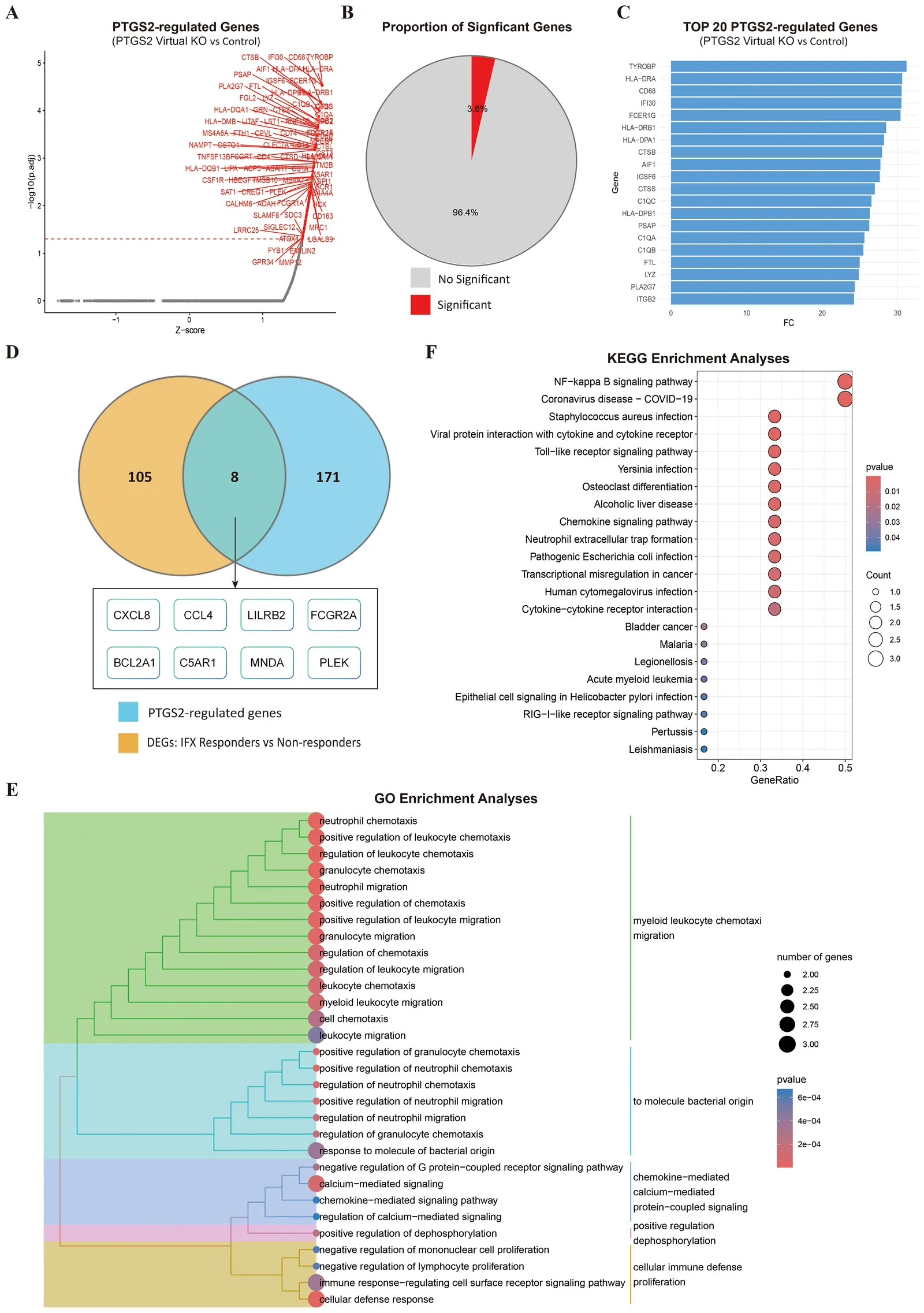
Transcriptional alterations induced by PTGS2 virtual knockout in the inflamed colon. **(A)** Volcano plot of genes affected by PTGS2 virtual knockout in the inflamed colon. **(B)** Pie chart showing the proportion of genes affected by PTGS2 virtual knockout among all detected background genes. **(C)** Bar plot of the top 20 genes most significantly affected by PTGS2 virtual knockout. **(D)** Venn diagram showing the overlap between *PTGS2*-regulated genes and IFX response-associated DEGs. **(E)** GO enrichment analysis of the overlapping genes shown in panel **(D)**. **(F)** KEGG enrichment analysis of the overlapping genes shown in panel **(D)**.

We next investigated whether *PTGS2* knockout influences the DEGs associated with primary IFX non-response. Intersection analysis of *PTGS2*-regulated genes (**Figure 3A**) and the DEGs identified between IFX responders and non-responders (**Figure 1B**) revealed eight overlapping genes, including *CXCL8*, *CCL4*, *LILRB2*, *FCGR2A*, *BCL2A1*, *C5AR1*, *MNDA*, and *PLEK* (**Figure 3D**). Notably, several of these shared genes, particularly CXCL8 and C5AR1, are well-established mediators of neutrophil recruitment and activation, suggesting a mechanistic link between *PTGS2* expression and the enhanced neutrophil infiltration observed in IFX non-responders.

To further characterize the biological functions of these overlapping genes, GO enrichment analysis was performed. The results demonstrated significant enrichment in myeloid leukocyte chemotaxis, neutrophil migration, and leukocyte recruitment-related biological processes (**Figure 3E**). At the Level 3 pathway scale, KEGG pathway analysis identified NF-κB signaling as the most significantly enriched term affected by *PTGS2* perturbation (**Figure 3F**).

The above results suggested that PTGS2 may functions as an important regulator of myeloid inflammatory programs in the inflamed colon and contribute to primary IFX non-response by promoting neutrophil recruitment through NF-κB-associated signaling pathways.

### 3.4 CD14^+^S100A9^+^ monocytes are a major cellular source of PTGS2 transcripts in the inflamed colon

We next investigated the cellular source of PTGS2 transcripts based on the single-cell RNA sequencing data derived from colonic biopsies of patients with UC. A total of 19 distinct cellular clusters were identified (**Figure 4A**). Using established lineage-specific marker genes, these clusters were classified into 11 cell subsets, including T cells, CD4^+^ T cells, B cells, epithelial cells, secretory B cells, macrophages, fibroblasts, endothelial cells, mast cells, monocytes, and neurons (**Figure 4B**). Analysis of cell-type proportions revealed substantial differences between inflamed and non-inflamed colonic tissues. Relative to non-inflamed tissues, inflamed colonic mucosa exhibited a marked reduction in epithelial cells, accompanied by a pronounced expansion of immune cell populations, particularly B cells, secretory B cells, and monocytes (**Figure 4C**).

**Figure 4:**
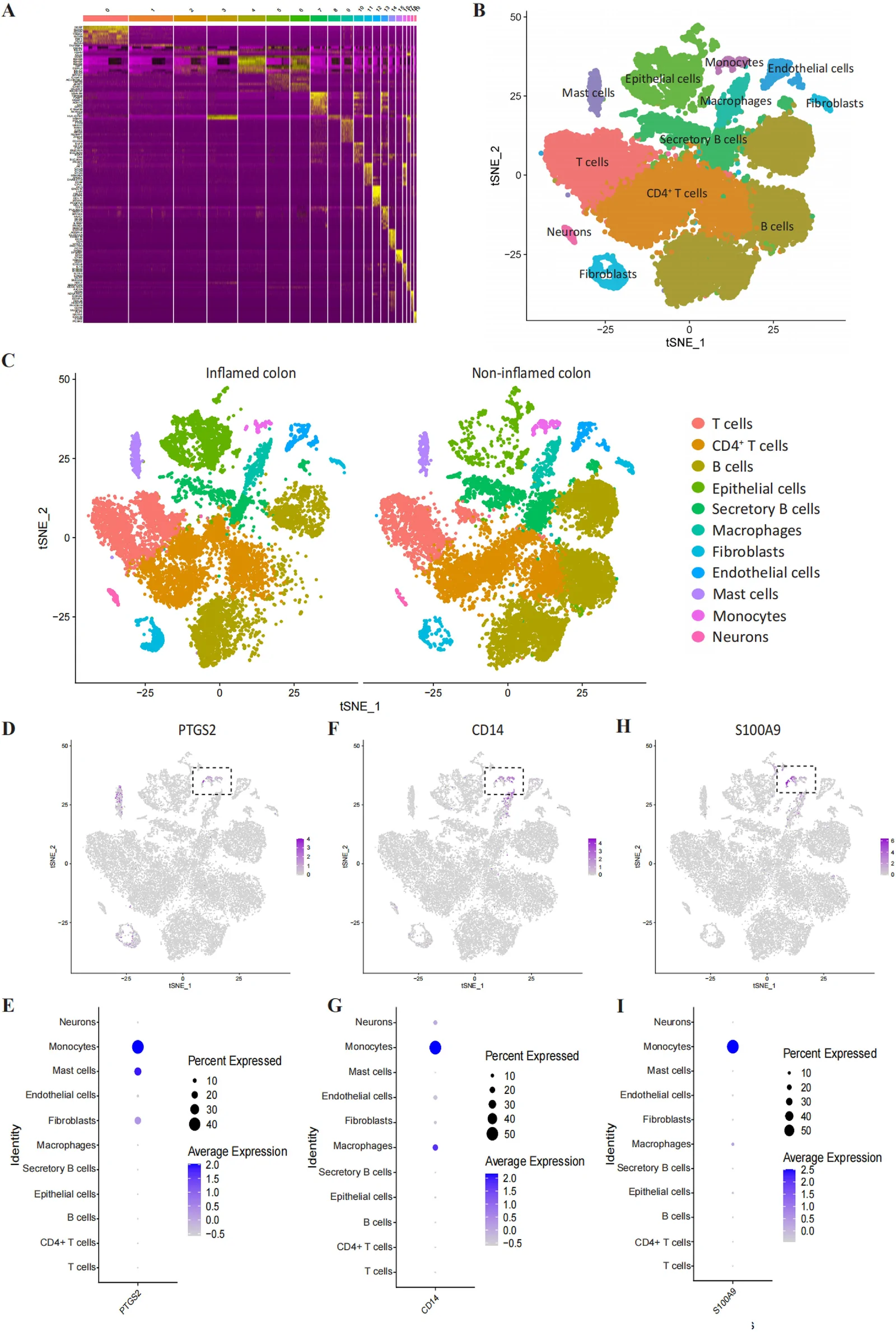
PTGS2 is predominantly expressed in CD14^+^S100A9^+^ inflammatory monocytes in the inflamed colon. **(A)** Heatmap of the 19 cell clusters identified based on their gene expression profiles. **(B)** t-SNE plot of the annotated cell populations. **(C)** Differential proportions of each cell population between inflamed and non-inflamed colonic tissues. **(D, E)** t-SNE feature plot **(D)** and dot plot **(E)** of *PTGS2* expression across different cell populations. **(F, G)** t-SNE feature plot **(F)** and dot plot **(G)** of *CD14* expression across different cell populations. **(H, I)** t-SNE feature plot **(H)** and dot plot **(I)** of *S100A9* expression across different cell populations.

The cellular distribution of *PTGS2* transcripts across all identified cell populations was subsequently investigated. Gene expression analysis based on t-SNE clustering revealed that PTGS2 transcripts were prominently expressed in a population of inflammatory monocytes (**Figures 4D, E**) characterized by high expression of *CD14* (**Figures 4F, G**) and *S100A9* (**Figures 4H, I**). Quantitative analysis further showed that monocytes exhibited the highest proportion of PTGS2-positive cells (41.41%), whereas smaller proportions of PTGS2-positive cells were observed in the other annotated cell populations (**Figures 4D, E**; **Supplementary Table 5**). Therefore, CD14^+^S100A9^+^ inflammatory monocytes represent a major cellular source of *PTGS2* transcripts in the inflamed colon. By integrating this finding with our bulk transcriptomic analyses showing the association of PTGS2 with primary non-response to IFX, it was hypothesized that this population of monocytes may play an important role in the enhanced neutrophil infiltration observed in IFX non-responders.

### 3.5 GQD primarily targets PTGS2 in primary IFX non-responders

Given the key role of *PTGS2* in primary IFX non-response, we further investigated whether Gegen Qinlian Decoction (GQD), a traditional herbal formula composed of *Radix Puerariae*, *Scutellariae Radix*, *Coptidis Rhizoma*, and *Liquorice*, could target PTGS2-associated molecular networks involved in IFX non-response.

A total of 146 bioactive compounds were identified from GQD (**Figure 5A**). Based on screening the TCMSP database, the potential target genes of these bioactive compounds were collected and integrated into a GQD-targeted gene set (**Supplementary Table 6**). Intersection analysis between GQD-target genes and the DEGs associated with IFX non-response (**Figures 1A, B**) identified 14 overlapping genes (**Figure 5B**), representing potential therapeutic targets through which GQD may modulate IFX responsiveness. Protein-protein interaction (PPI) analysis further revealed a highly interconnected interaction network among the 14 overlapping genes (**Figure 5C**). Notably, five genes, including PTGS2, exhibited direct interactions with all other overlapping genes within the network, indicating their potential roles as central regulatory nodes (**Figure 5D**).

**Figure 5:**
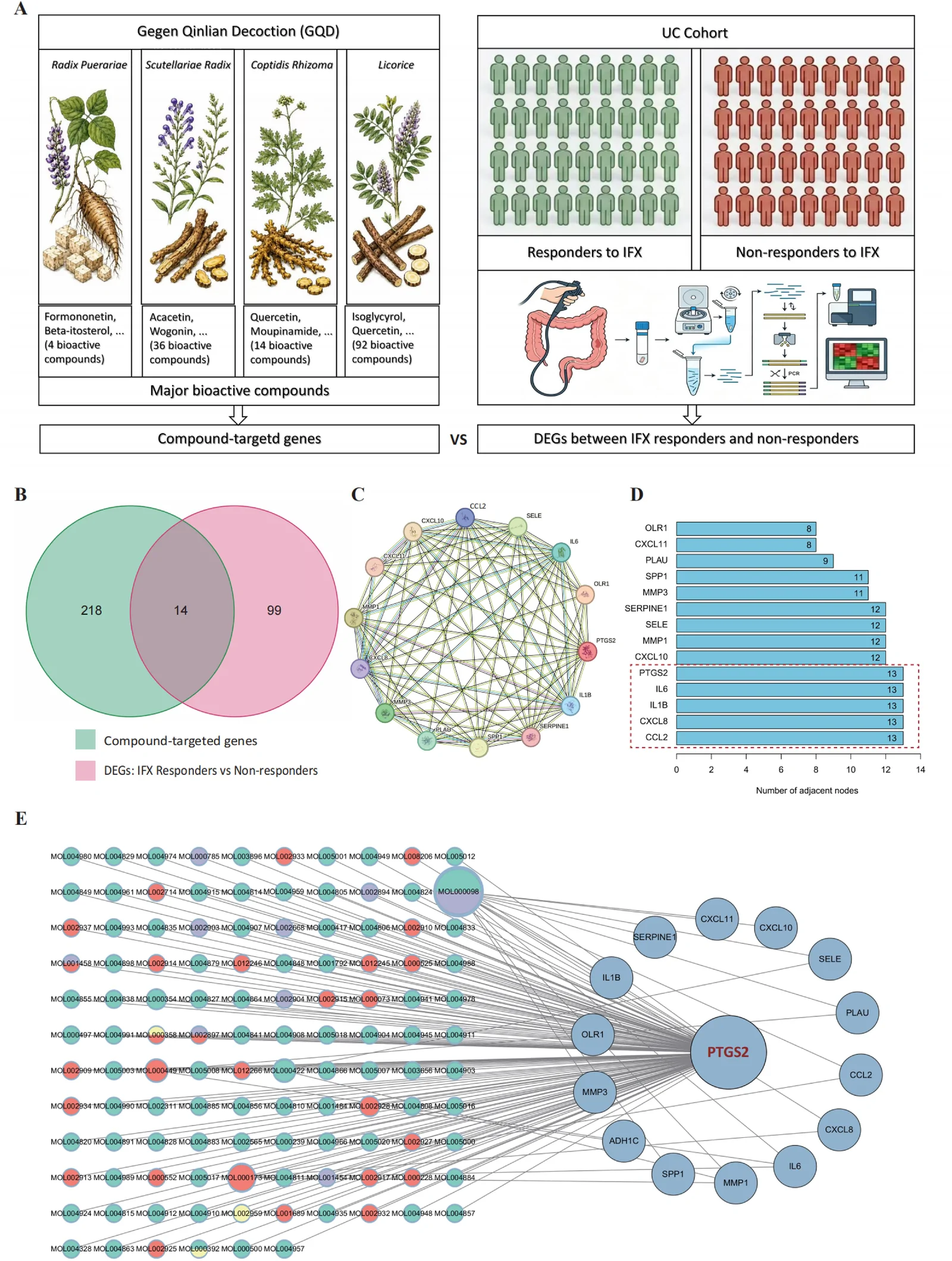
PTGS2 is a core GQD-targeted gene associated with primary IFX non-response. **(A)** Schematic workflow illustrating the identification of overlapping genes between GQD-targeted genes and IFX response-associated DEGs. **(B)** Venn diagram showing the overlap between GQD-targeted genes and IFX response-associated DEGs. **(C)** Protein-protein interaction (PPI) network of the 14 overlapping genes. **(D)** Bar plot showing the degree (number of adjacent nodes) of the 14 overlapping genes in the PPI network. **(E)** Cytoscape network illustrating the interactions between GQD bioactive compounds and the 14 overlapping genes, with node size representing degree.

To further identify the hub gene of these candidate targets, a compound-target interaction network was constructed and analyzed. Network topology analysis revealed substantial differences in connectivity within the 14 overlapping genes and GQD-derived bioactive compounds (**Figure 5E**). Among all candidate targets, PTGS2 exhibited the highest degree of connectivity and was linked to the largest number of bioactive compounds present in GQD. These observations suggest that PTGS2 is the principal hub target of GQD, highlighting the therapeutic potential of GQD for improving IFX responsiveness in UC.

### 3.6 Quercetin serves as the representative PTGS2-targeting constituent of GQD

Having identified PTGS2 as the principal hub target of GQD, we next sought to determine which bioactive constituent predominantly contributes to PTGS2-targeted regulation. Based on the compound-target interaction network (**Figure 5E**), all compounds linked to the 14 overlapping genes were ranked according to their degree values. The six top-ranked bioactive compounds, including quercetin (MOL000098), stigmasterol (MOL000449), kaempferol (MOL000422), isorhamnetin (MOL000354), oroxylin A (MOL002928) and wogonin (MOL000173), were selected for further analysis. These bioactive compounds were all predicted to target PTGS2. Among these candidates, quercetin (MOL000098) exhibited the highest degree value within the network, indicating the strongest connectivity and suggesting a superior potential for coordinated regulation of genes associated with IFX non-response (**Figures 5E**, **6A**).

**Figure 6:**
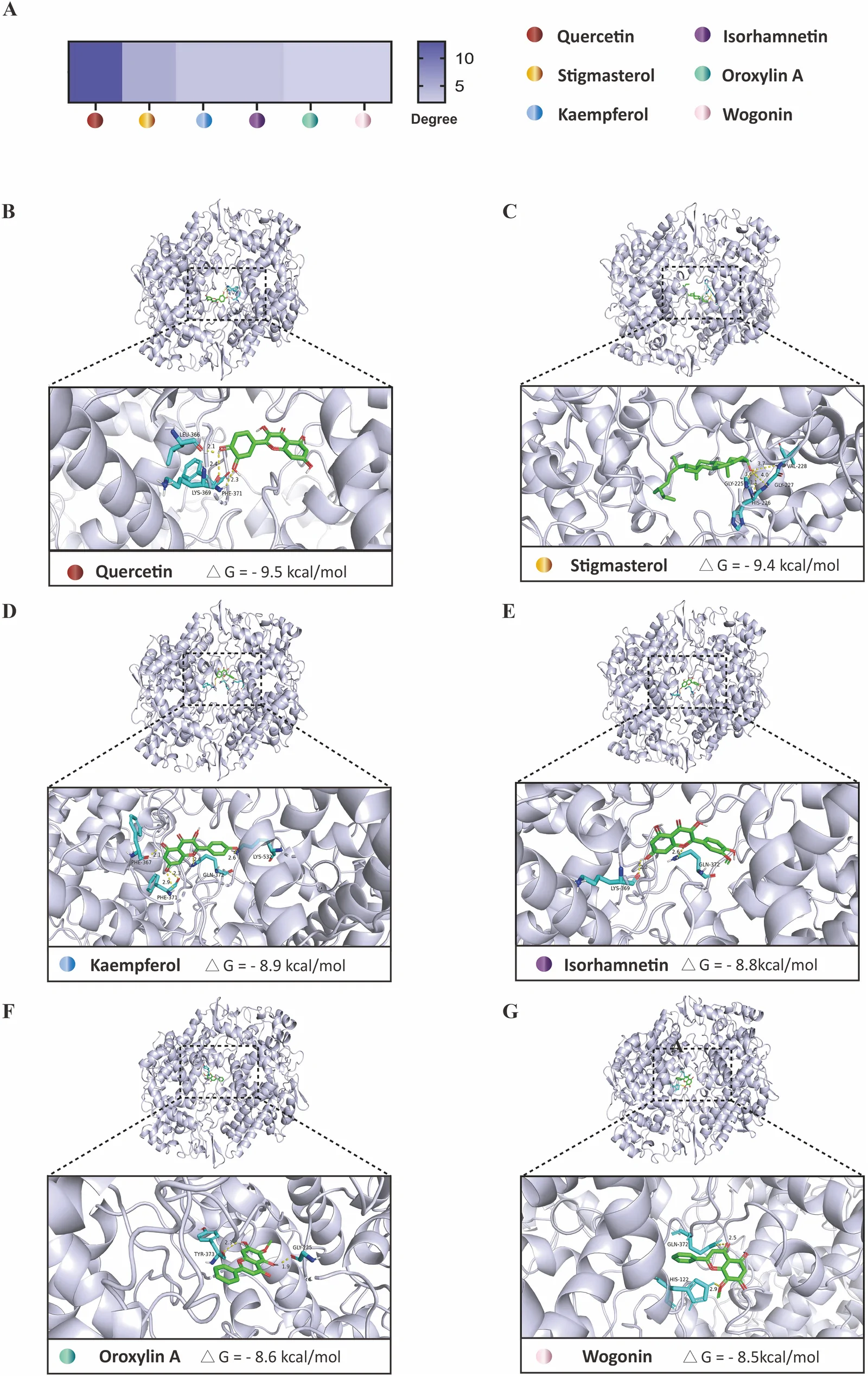
Molecular docking identifies quercetin as the bioactive compound with the strongest binding affinity toward PTGS2. **(A)** Heatmap showing the top six GQD bioactive compounds ranked by degree in the network pharmacology analysis. **(B-G)** Schematic representations of the molecular docking between PTGS2 and the top six GQD bioactive compounds: quercetin **(B)**, stigmasterol **(C)**, kaempferol **(D)**, isorhamnetin **(E)**, oroxylin A **(F)**, and wogonin **(G)**.

To further quantify the binding interactions between candidate compounds and PTGS2, molecular docking analysis was performed. Within the catalytic pocket located in the central region of the PTGS2 three-dimensional crystal structure, quercetin displayed the most favorable binding configuration compared with all other candidate compounds (**Figure 6B**). This interaction was supported by the lowest binding free energy (ΔG = -9.5 kcal/mol), together with more hydrogen bonds and shorter hydrogen-bond distances than those observed for the other compounds (**Figures 6C–G**).

Therefore, the compound-target network analysis and molecular docking results consistently identified quercetin as the most promising PTGS2-targeting constituent of GQD. These findings suggest that quercetin may serve as the representative bioactive component contributing to the therapeutic effects of GQD on PTGS2-associated inflammation and IFX responsiveness in UC.

### 3.7 Quercetin attenuates inflammatory monocyte-mediated neutrophil recruitment by suppressing the PTGS2/NF-κB/CXCL8 axis

Given that quercetin exhibited the strongest binding affinity toward PTGS2 among the major bioactive compounds identified in GQD, we next investigated whether this interaction could modulate PTGS2 expression and related inflammatory signaling. As described above, PTGS2 was predominantly expressed in inflammatory monocytes within the colonic mucosa. We therefore used undifferentiated THP-1 cells as an *in vitro* monocyte model for subsequent mechanistic studies.

Under LPS stimulation, PTGS2 expression was markedly increased in THP-1 cells, whereas quercetin treatment significantly reduced this induction (**Figures 7A, B**). Concomitant with the reduction in PTGS2 expression, NF-κB signaling was also suppressed in LPS-stimulated THP-1 monocytes, as evidenced by decreased phosphorylated p65 levels (**Figures 7A, C**) and a reduced p-p65/p65 ratio (**Figures 7A, D**). CXCL8, a well-established downstream effector of NF-κB signaling, was also significantly reduced by quercetin (**Figures 7A, E**). Consistent with these molecular changes, quercetin significantly attenuated the ability of LPS-stimulated THP-1 cells to induce neutrophil chemotaxis, as assessed using DMSO-differentiated HL-60 cells in a Transwell chemotaxis assay (**Figures 7F, G**). Notably, the concentration of quercetin used in these experiments (25 μM) had no significant effect on THP-1 cell viability (**Figure 7H**), further supporting that the observed inhibitory effects were not attributable to cytotoxicity but were more likely associated with the modulation of PTGS2-related inflammatory signaling by quercetin.

**Figure 7:**
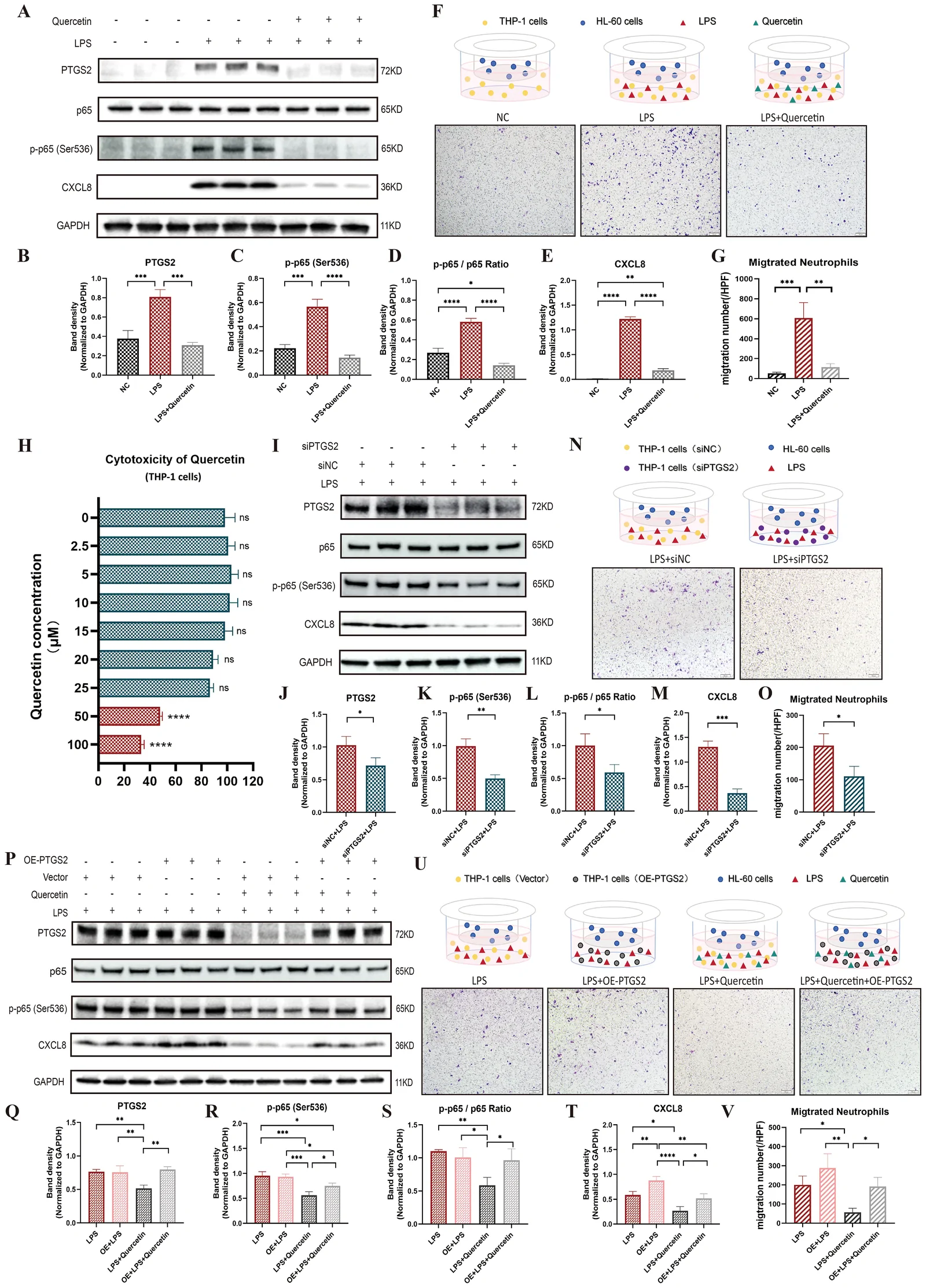
Quercetin suppresses activation of the PTGS2/NF-κB/CXCL8 axis and attenuates THP-1 monocyte-induced neutrophil chemotaxis. **(A)** Representative Western blot bands of PTGS2, NF-κB p65, phospho-p65 (p-p65), and CXCL8 in LPS-stimulated THP-1 monocytes with or without quercetin treatment. **(B-E)** Quantification of the protein expression levels of PTGS2 **(B)**, p-p65 **(C)**, the p-p65/p65 ratio **(D)**, and CXCL8 **(E)**. **(F, G)** Representative images **(F)** and quantitative analysis **(G)** of migrated HL-60 cells per high-power field in the Transwell chemotaxis assay under the indicated treatment conditions. **(H)** Cell viability of THP-1 cells following treatment with quercetin, as assessed by the CCK-8 assay. **(I)** Representative Western blot bands of PTGS2, NF-κB p65, p-p65, and CXCL8 in LPS-stimulated THP-1 cells transfected with PTGS2-specific siRNA (siPTGS2) or negative-control siRNA (si-NC). **(J-M)** Quantification of PTGS2 **(J)**, p-p65 **(K)**, the p-p65/p65 ratio **(L)**, and CXCL8 **(M)**. **(N, O)** Representative images **(N)** and quantitative analysis **(O)** of migrated HL-60 cells per high-power field in the Transwell chemotaxis assay following PTGS2 knockdown. **(P)** Representative Western blot bands of PTGS2, NF-κB p65, p-p65, and CXCL8 in THP-1 cells transfected with the PTGS2 overexpression plasmid (OE-PTGS2) or empty vector and treated with LPS and/or quercetin as indicated. **(Q-T)** Quantification of PTGS2 **(Q)**, p-p65 **(R)**, the p-p65/p65 ratio **(S)**, and CXCL8 **(T)**. **(U, V)** Representative images **(U)** and quantitative analysis **(V)** of migrated HL-60 cells per high-power field in the Transwell chemotaxis assay under the indicated PTGS2 overexpression and quercetin treatment conditions. Data are presented as mean ± SEM. Based on data distribution and homogeneity of variance, two-group comparisons were performed using an unpaired Student’s t-test or Mann-Whitney U test, while multiple-group comparisons were performed using one-way ANOVA followed by Tukey’s *post hoc* test or the Kruskal-Wallis test followed by Dunn’s *post hoc* test. Statistical significance was defined as *P < 0.05, **P < 0.01, ***P < 0.001, and ****P < 0.0001.

These findings are consistent with our virtual knockout analysis (**Figure 4F**), which identified NF-κB signaling as the most significantly perturbed pathway associated with PTGS2 knockout. To further determine whether NF-κB signaling is downstream of PTGS2, we knocked down PTGS2 in THP-1 cells using small interfering RNA (siRNA) (**Figures 7I, J**). It was observed that PTGS2 knockdown significantly reduced p-p65 levels (**Figures 7I, K**), the p-p65/p65 ratio (**Figures 7I, L**), and CXCL8 expression (**Figures 7I, M**) in LPS-stimulated THP-1 cells. These molecular changes were accompanied by an obvious reduction in the chemotactic activity of THP-1 cells toward HL-60 cells (**Figures 7N, O**).

We further performed a rescue experiment based on exogenous PTGS2 overexpression (OE-PTGS2) (**Figure 7P**). Although quercetin reduced the LPS-induced increase in PTGS2 expression (**Figures 7P, Q**), suppressed NF-κB signaling (**Figures 7P, R-T**) and attenuated chemotaxis toward HL-60 cells (**Figures 7U, V**), exogenous PTGS2 partially restored these changes in the presence of quercetin. Collectively, these results indicate that NF-κB signaling in inflammatory monocytes is, at least in part, dependent on PTGS2 expression. Considering that inflammatory monocytes represent one of the major cellular sources of CXCL8 within inflamed tissues (28), these findings suggest that quercetin may attenuate neutrophil chemotaxis in the inflamed colon by suppressing the PTGS2/NF-κB/CXCL8 signaling in colonic inflammatory monocytes.

### 3.8 GQD enhances the therapeutic efficacy of anti-TNF-α (IFX) treatment in experimental colitis

The effect of GQD on the therapeutic efficacy of IFX was further assessed in DSS-induced colitis in mice (**Figure 8A**). Monotherapy with either the mouse-specific anti-TNF-α antibody (used as a surrogate for IFX) or GQD failed to significantly alleviate the severity of experimental colitis. In contrast, their combined administration markedly ameliorated the disease severity, as evidenced by reduced body weight loss (**Figure 8B**), lower disease activity index (DAI) (**Figure 8C**), increased colon length (**Figures 8D, E**), and improved histological activity index (HAI) (**Figures 8F, G**), supporting the ability of GQD to enhance the therapeutic efficacy of anti-TNF-α treatment in experimental colitis.

**Figure 8:**
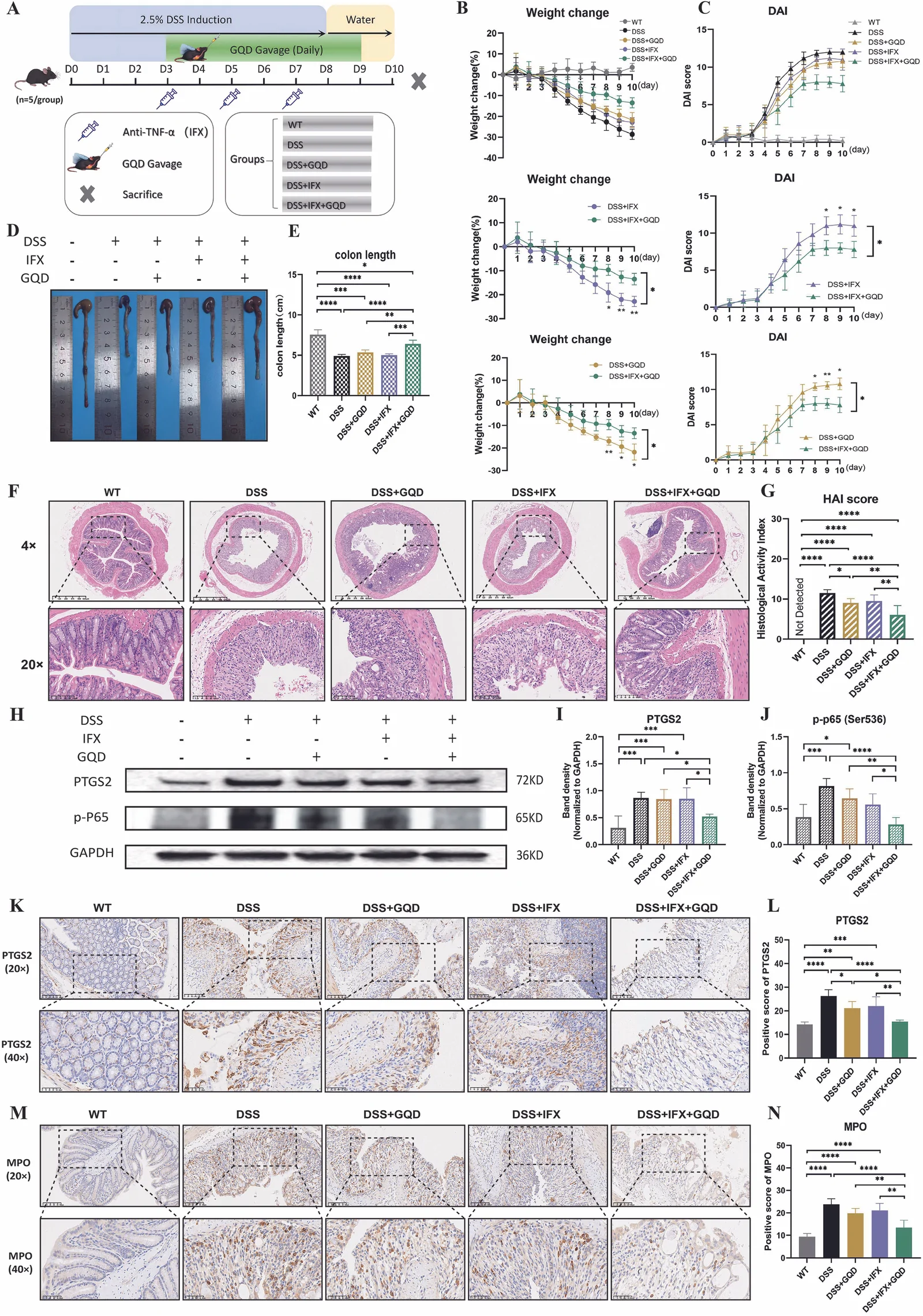
GQD improves IFX responsiveness in experimental colitis. **(A)** Schematic illustration of the experimental design and treatment protocol. **(B-C)** Body weight changes **(B)** and DAI scores **(C)** of mice from the indicated treatment groups. Top, all groups; middle, DSS+IFX vs. DSS+IFX+GQD; bottom, DSS+GQD vs. DSS+IFX+GQD. **(D-E)** Representative images **(D)** and quantification of colon length **(E)** in mice from the indicated treatment groups. **(F-G)** Representative hematoxylin and eosin (H&E)-stained images of colonic tissues **(F)** and HAI scores **(G)** of mice in the indicated treatment groups. **(H-J)** Representative Western blot images of PTGS2 and phosphorylated NF-κB p65 (p-p65) in colonic tissues **(H)** and corresponding quantification of PTGS2 **(I)** and p-p65 **(J)** protein levels. **(K, L)** Representative immunohistochemical images of colonic PTGS2 expression **(K)** and corresponding quantitative analysis **(L)** in the indicated treatment groups. **(M, N)** Representative immunohistochemical images of colonic MPO expression **(M)** and corresponding quantitative analysis **(N)** in the indicated treatment groups. Data are presented as mean ± SEM. Based on data distribution and homogeneity of variance, multiple-group comparisons were performed using one-way ANOVA followed by Tukey’s *post hoc* test or the Kruskal-Wallis test followed by Dunn’s *post hoc* test. Body weight and DAI over time were analyzed by two-way ANOVA followed by Bonferroni’s multiple-comparison test. Statistical significance was defined as *P < 0.05, **P < 0.01, ***P < 0.001, and ****P < 0.0001.

At the molecular level, the protein levels of PTGS2 and phosphorylated NF-κB p65 (p-p65) were markedly increased in the inflamed colonic tissues of DSS-challenged mice (**Figure 8H**). Notably, compared with either anti-TNF-α (IFX) or GQD monotherapy, combined GQD and anti-TNF-α (IFX) treatment significantly reduced the protein levels of PTGS2 (**Figures 8H, I**) and p-p65 (**Figures 8H, J**), reflecting suppression of NF-κB signaling. Immunohistochemical staining further revealed reduced PTGS2 expression in inflammatory cell-infiltrated areas of the colonic mucosa following combined treatment (**Figures 8K, L**). Consistent with the *in vitro* findings, reduced PTGS2 expression was accompanied by a marked reduction in neutrophil infiltration within the inflamed colonic mucosa. This was evidenced by the reduced expression of myeloperoxidase (MPO), a well-established neutrophil-specific marker, in mice receiving combined GQD and anti-TNF-α (IFX) treatment (**Figures 8M, N**). Taken together, these results demonstrate that GQD treatment contributed to reducing PTGS2 expression, suppressing NF-κB signaling, and decreasing neutrophil infiltration in the inflamed colon, thereby enhancing the therapeutic efficacy of anti-TNF-α (IFX) in experimental colitis.

## 4 Discussion

Primary non-response to IFX remains a major unmet clinical challenge during the treatment of UC. In this study, we identified a previously unrecognized role of inflammatory monocyte-derived PTGS2 in impaired response to IFX, potentially through promoting neutrophil recruitment to the inflamed colon. Moreover, we further demonstrated that the traditional Chinese herbal formula GQD represents a promising therapeutic strategy for enhancing the therapeutic efficacy of IFX by targeting PTGS2-associated neutrophil recruitment. These findings provide new mechanistic insights into the molecular mechanisms underlying impaired primary response to IFX, and support the potential of GQD as an adjunctive therapeutic strategy targeting PTGS2 to enhance IFX efficacy in UC.

As a key effector cell type in intestinal inflammation, neutrophils play an important role in the pathogenesis of UC. Activated neutrophils amplify mucosal inflammation through the release of reactive oxygen species (29), proteolytic enzymes (30), and neutrophil extracellular traps (31–33), thereby promoting epithelial injury and sustaining inflammatory signaling within the intestinal microenvironment. Notably, accumulating evidence suggests that excessive neutrophil infiltration may contribute to primary IFX non-response in UC. Neutrophil-related gene signatures (34), autoantibodies (35) and ratios of routine clinical parameters (36) have been identified as predictors of poor response to IFX. However, the relationship between baseline colonic neutrophil infiltration and primary IFX non-response has not been directly investigated, and the molecular mechanisms responsible for neutrophil accumulation in non-responders remain largely unknown. By integrating transcriptomic analyses and validation in clinical specimens, our study directly demonstrates that, despite comparable baseline colonic inflammatory severity, patients with more baseline colonic neutrophil infiltration exhibit a poorer primary response to IFX. Extensive baseline neutrophil infiltration in the colonic mucosa may reflect an inflammatory microenvironment that is insufficiently controlled by TNF-α blockade, thereby contributing to primary IFX non-response. Notably, this difference in neutrophil infiltration was closely associated with PTGS2 expression in the inflamed colon, suggesting that PTGS2 may participate in shaping the inflammatory microenvironment related to IFX non-response.

PTGS2, an inducible enzyme that catalyzes the conversion of arachidonic acid into prostaglandins, plays a central role in regulating inflammatory responses during intestinal inflammation (37). Elevated PTGS2 expression has been observed in the inflamed mucosa of patients with UC and is generally associated with enhanced inflammatory responses (38–40). A limited number of studies have reported an association between PTGS2 expression and neutrophil infiltration in several extraintestinal tissue types (41, 42), which is consistent with our findings. However, the specific cellular source driving this process and the underlying mechanisms remain largely unclear. According to our single-cell transcriptomic analyses, PTGS2 was found to be predominantly expressed in inflammatory monocytes within inflamed colonic tissues from patients with UC. These findings are in agreement with a recent single-cell study demonstrating preferential PTGS2 expression in myeloid cells within inflamed human colonic tissues (43). Notably, our analysis further refined the cellular origin of PTGS2 by identifying infiltrating CD14^+^S100A9^+^inflammatory monocytes as the cell population with the highest proportion of PTGS2-positive cells. This observation is of interest because inflammatory monocytes are increasingly recognized as major orchestrators of leukocyte recruitment in inflamed tissues (44–46).

The virtual knockout analysis employed in this study provided further mechanistic insight into how PTGS2 may contribute to neutrophil recruitment, identifying NF-κB signaling as the most significantly perturbed pathway following PTGS2 knockout. This computational finding was further supported by bidirectional experimental manipulation of PTGS2 in monocytes. PTGS2 knockdown markedly reduced NF-κB signaling, CXCL8 expression, and the chemotactic activity of inflammatory monocytes toward neutrophils. Conversely, exogenous PTGS2 overexpression markedly restored NF-κB signaling, CXCL8 expression, and neutrophil chemotaxis in the presence of quercetin. These findings are also consistent with previous studies showing that PTGS2 is involved in macrophage inflammatory responses and is closely associated with NF-κB signaling (47, 48). Notably, PTGS2 overexpression exerted relatively modest effects on downstream signaling and neutrophil chemotaxis under LPS stimulation alone. This may be attributed to the strong induction of endogenous PTGS2 by LPS, resulting in a high inflammatory background that could limit the additional effect of exogenous PTGS2 overexpression. Nevertheless, under conditions of quercetin-induced PTGS2 downregulation, exogenous PTGS2 overexpression exhibited a marked restorative effect on NF-κB/CXCL8 signaling and neutrophil chemotaxis. Collectively, these findings support PTGS2 as an upstream regulator of the NF-κB/CXCL8 axis and suggest that PTGS2 may promote neutrophil recruitment through this signaling pathway. Given that inflammatory monocytes represent one of the major cellular sources of CXCL8 within inflamed tissues, the above results further indicate a functional role for monocyte-derived PTGS2 in promoting neutrophil recruitment during intestinal inflammation.

Identifying an effective therapeutic strategy to target PTGS2 was another objective of this study. Although several selective PTGS2 inhibitors, including celecoxib, etoricoxib, and parecoxib, are clinically available, their therapeutic applications are primarily limited to the management of pain and inflammation in disorders such as osteoarthritis (49, 50) and rheumatoid arthritis (51, 52), and none has been approved for the treatment of UC. Furthermore, conventional PTGS2 inhibitors are designed to target PTGS2 through a single active compound, which may impose certain therapeutic limitations. In contrast, traditional Chinese herbal medicine, which typically acts on multiple molecular targets through diverse bioactive constituents, has increasingly been recognized as a valuable source of novel therapeutic agents. Among these, GQD, a traditional Chinese herbal formula recommended by the Board of Specialty Committee of Digestive System Disease of the World Federation of Chinese Medicine Societies (53), has been used in the treatment of UC for years. Our findings demonstrated that GQD suppressed PTGS2 expression, NF-κB activation, and neutrophil infiltration in experimental colitis, suggesting PTGS2-associated inflammatory signaling as a potential mechanism underlying GQD efficacy.

Specifically, Gegen Qinlian Decoction (GQD), composed of *Radix Puerariae*, *Scutellariae Radix*, *Coptidis Rhizoma*, and *Liquorice*, is a classical traditional Chinese herbal formula that has been used for the treatment of gastrointestinal inflammatory disorders for centuries (25, 54). Previous studies have demonstrated that GQD exerts therapeutic effects through multiple mechanisms, including restoration of intestinal barrier integrity (55), modulation of gut microbial composition (56) and metabolites (57, 58), and regulation of T-cell differentiation (54, 59, 60) and immune homeostasis (61). Our findings further expand these mechanistic insights. In this study, GQD, through a multi-targeted mechanism predominantly involving quercetin, reduced PTGS2 expression, thereby downregulating the NF-κB/CXCL8 signaling and reducing neutrophil recruitment. This provides a novel and translational solution to enhance IFX responsiveness in UC.

Despite these promising findings, this study has several limitations:

- The clinical validation cohort was derived from a single center and remained relatively small, which may limit the statistical power and generalizability of our findings. Further validation in larger, independent, prospective cohorts is therefore warranted.
- The therapeutic validation of GQD as an adjunct to IFX is currently limited to cell and experimental animal models. Although our findings support the potential clinical translation of this combination strategy, its therapeutic efficacy and clinical applicability remain to be validated in prospective clinical studies. Future studies are therefore needed to optimize the treatment regimen and evaluate the long-term efficacy and safety of this combination strategy.
- The single-cell RNA-sequencing analysis was based on a relatively limited number of samples. Although this analysis provided cell-level resolution of PTGS2 expression across different cellular populations, validation in larger independent single-cell datasets would further strengthen the generalizability of these findings.
- Given the complex and multifactorial determinants of primary non-response to IFX, there is currently no well-established and reproducible animal model that fully recapitulates this clinical phenotype. Indeed, insufficient IFX exposure resulting from factors such as early immunogenicity (62), accelerated drug clearance (63), high inflammatory burden (64), and intestinal drug loss (65) represents an important contributor to primary treatment failure, providing a rationale for the use of low-dose anti-TNF-α treatment in our combination experiments. Nevertheless, this low-dose experimental model still cannot fully reproduce the biological complexity of primary IFX non-response in patients with UC. Therefore, these limitations should be considered when translating the present experimental findings to the clinical setting.
- Although our *in vitro* and *in vivo* findings consistently support the involvement of PTGS2 in the therapeutic effects of GQD, future studies using PTGS2-specific genetic manipulation *in vivo* would provide additional evidence to further substantiate its mechanistic role.

## 5 Conclusion

Collectively, this study provides novel insights into the mechanisms underlying primary non-response (PNR) to IFX in UC. We identified PTGS2 as a key molecular feature of PNR and further localized its expression predominantly to CD14^+^S100A9^+^ inflammatory monocytes in inflamed colonic tissues. Mechanistically, PTGS2 promotes neutrophil recruitment through activating the NF-κB/CXCL8 signaling, thereby contributing to impaired responsiveness to IFX. Moreover, GQD suppressed PTGS2/NF-κB signaling, attenuated neutrophil recruitment, and ultimately improved the efficacy of anti-TNF-α(IFX) treatment in experimental colitis. These findings identify PTGS2 as a promising therapeutic target for overcoming primary IFX non-response and highlight the translational potential of GQD as a therapeutic strategy to enhance IFX responsiveness.

## Data Availability

The original contributions presented in the study are included in the article/**Supplementary Material**. Further inquiries can be directed to the corresponding authors.
