# Supplementary material for: Overcoming primary non-response to infliximab in ulcerative colitis: monocyte-derived PTGS2 as a therapeutic target
Source: Front Immunol. 2026 Oct 20;17:1957588. doi: 10.3389/fimmu.2026.1957588 (PMC13727455; doi:10.3389/fimmu.2026.1957588)
Supplement: Supplementary file 1 [file SupplementaryFile1.docx]

**Supplementary Materials**

**Supplementary Tables**

**Supplementary Table 1: Baseline Characteristics of Infliximab Responders and Non-responders with Ulcerative Colitis**

| **Characteristic** | **Non-responders**  **(n=21)** | **Responders (n=23)** | **P value** |
| --- | --- | --- | --- |
| Age（years） | 45 (31-55) | 50 (41-54) | 0.8568 |
| Sex（male/female） | 8/13 (38.1%/61.9%) | 10/13 (43.5%/56.5%) | 0.22 |
| Disease duration（years） | 4 (2.5-4.0) | 3 (2.0-6.0) | 0.8925 |
| Disease extent |  |  | 0.6944 |
| E1 | 2 (9.5%) | 2 (8.7%) |  |
| E2 | 7 (33.3%) | 10 (43.5%) |  |
| E3 | 12 (57.1%) | 11 (47.8%) |  |
| Baseline MMDAI | 8.0 (6.0-11.0) | 9.0 (6.0-10.0) | 0.5893 |
| Baseline MES | 2.0 (2.0-3.0) | 2.0 (2.0-3.0) | 0.5406 |
| IFX induction dose | 5 mg/kg | 5 mg/kg | - |

Notes: Continuous variables are presented as median (IQR), and categorical variables as n (%). The Mann-Whitney U test was used for continuous variables, and Pearson’s χ² test was used for categorical variables. MMDAI, Modified Mayo Disease Activity Index; MES, Mayo Endoscopic Score; IFX, infliximab; R, primary responder; NR, primary non-responder; IQR, interquartile range.

**Supplementary Table 2: Quality control information for the dispensing granules used in Gegen Qinlian Decoction (GQD)**

| **Component** | **Batch No.** | **Granule specification** | **Crude-herb equivalent** |
| --- | --- | --- | --- |
| *Radix Puerariae* | 25040471 | 1.0g granules | 2.5g Crude-herb equivalent |
| *Scutellariae Radix* | 25100181 | 1.0g granules | 2.2g Crude-herb equivalent |
| *Coptidis Rhizoma* | 26010771 | 1.0g granules | 4.5g Crude-herb equivalent |
| *Liquorice* | 25110111 | 1.0g granules | 3.0g Crude-herb equivalent |

**Supplementary Table 3: Baseline differentially expressed genes in colonic tissues between primary infliximab responders and non-responders with ulcerative colitis**

| **Genes** | **logFC** | **Average**  **Expression** | **t statistic** | **P Value** | **Adjusted P value** | **B statistic** |
| --- | --- | --- | --- | --- | --- | --- |
| CHP2 | -1.488412754 | 7.483852873 | -4.170108043 | 8.498E-05 | 2.26E-03 | 1.302537631 |
| BEST4 | -1.163195299 | 6.452181946 | -3.126030603 | 2.569E-03 | 2.26E-02 | -1.809662766 |
| GUCA2B | -1.099534129 | 8.106223451 | -3.336741254 | 1.353E-03 | 1.46E-02 | -1.23143992 |
| ADH1C | -1.080280652 | 8.52298508 | -3.373905639 | 1.205E-03 | 1.35E-02 | -1.126625337 |
| RETNLB | -1.079068363 | 8.326645036 | -3.20766364 | 2.010E-03 | 1.91E-02 | -1.588936631 |
| HSD11B2 | -1.029201402 | 9.852427123 | -4.585300338 | 1.902E-05 | 9.69E-04 | 2.690372096 |
| GUCA2A | -1.006761265 | 9.574567301 | -3.238073023 | 1.832E-03 | 1.79E-02 | -1.50564041 |
| PTGFR | 1.000964158 | 5.80235472 | 5.198464232 | 1.858E-06 | 2.27E-04 | 4.859862349 |
| FCN1 | 1.004753095 | 7.581523671 | 4.67466436 | 1.366E-05 | 7.87E-04 | 2.998289497 |
| TLR8 | 1.005921862 | 7.108182567 | 5.599228291 | 3.822E-07 | 8.86E-05 | 6.34010914 |
| PLAU | 1.00760747 | 9.530223093 | 5.507347105 | 5.512E-07 | 1.07E-04 | 5.997030531 |
| LILRB3 | 1.008029387 | 8.007293786 | 5.289414916 | 1.302E-06 | 1.88E-04 | 5.191965944 |
| HK3 | 1.010813627 | 5.654792001 | 3.886003041 | 2.268E-04 | 4.38E-03 | 0.397808443 |
| CLEC4D | 1.012784973 | 5.939086159 | 5.533451315 | 4.969E-07 | 1.03E-04 | 6.094291001 |
| COL8A1 | 1.014352545 | 5.550150891 | 5.149052326 | 2.251E-06 | 2.53E-04 | 4.680453684 |
| MCEMP1 | 1.014767785 | 7.318854324 | 3.907338732 | 2.110E-04 | 4.18E-03 | 0.464376089 |
| FGF2 | 1.021403213 | 7.211808774 | 5.182047979 | 1.980E-06 | 2.35E-04 | 4.800176034 |
| PTX3 | 1.024040981 | 6.390659881 | 4.804952168 | 8.386E-06 | 5.91E-04 | 3.452566573 |
| LILRB1 | 1.027328613 | 7.312736507 | 5.202455692 | 1.829E-06 | 2.27E-04 | 4.874386577 |
| SPOCK1 | 1.02944319 | 6.441707379 | 5.662087312 | 2.972E-07 | 7.85E-05 | 6.575987678 |
| MGAM | 1.033023864 | 6.799017119 | 3.337244315 | 1.350E-03 | 1.46E-02 | -1.230026688 |
| PTHLH | 1.035128704 | 5.492365433 | 5.342206117 | 1.059E-06 | 1.71E-04 | 5.385808825 |
| LY96 | 1.038222154 | 8.968932331 | 5.704446977 | 2.507E-07 | 7.50E-05 | 6.735457217 |
| CYR61 | 1.052237698 | 8.803012197 | 4.034213374 | 1.365E-04 | 3.09E-03 | 0.864913899 |
| NLRP3 | 1.055796989 | 5.692646954 | 4.492612028 | 2.672E-05 | 1.20E-03 | 2.374296708 |
| SAMSN1 | 1.056506099 | 8.225167606 | 5.884187478 | 1.213E-07 | 5.56E-05 | 7.41647798 |
| OLR1 | 1.060638726 | 6.272454614 | 4.613864173 | 1.711E-05 | 9.22E-04 | 2.788459587 |
| IL7R | 1.06570915 | 9.390765297 | 5.451099643 | 6.890E-07 | 1.28E-04 | 5.78804579 |
| HSD11B1 | 1.066430821 | 6.658630793 | 6.455809735 | 1.157E-08 | 1.85E-05 | 9.622028202 |
| TLR1 | 1.071402407 | 7.110102416 | 5.983319748 | 8.103E-08 | 4.92E-05 | 7.794908228 |
| CCL8 | 1.072066498 | 7.006375406 | 4.501933476 | 2.583E-05 | 1.17E-03 | 2.405928229 |
| PDE4B | 1.074762045 | 8.183943999 | 5.8761812 | 1.253E-07 | 5.56E-05 | 7.385999159 |
| ACSL1 | 1.076084579 | 9.481478054 | 4.65058151 | 1.494E-05 | 8.43E-04 | 2.915007937 |
| CCL4 | 1.078485976 | 8.198011793 | 4.794920697 | 8.709E-06 | 6.09E-04 | 3.417370822 |
| CCDC3 | 1.083296232 | 7.516319621 | 4.386148942 | 3.934E-05 | 1.48E-03 | 2.015556462 |
| ACSL4 | 1.0908447 | 8.233559299 | 6.511636543 | 9.174E-09 | 1.62E-05 | 9.840104618 |
| CYP24A1 | 1.092980955 | 4.925500773 | 4.938286689 | 5.060E-06 | 4.48E-04 | 3.923710492 |
| CXCL10 | 1.095835642 | 9.682343996 | 4.167515586 | 8.576E-05 | 2.26E-03 | 1.294108339 |
| SPP1 | 1.10037312 | 8.317865854 | 3.797932291 | 3.052E-04 | 5.32E-03 | 0.125493943 |
| MMP10 | 1.104644111 | 8.860569984 | 4.394390465 | 3.818E-05 | 1.47E-03 | 2.043158536 |
| NCF2 | 1.106905005 | 8.590105731 | 4.90074838 | 5.837E-06 | 4.76E-04 | 3.790449541 |
| C5AR1 | 1.118025898 | 8.132881839 | 5.277626396 | 1.364E-06 | 1.90E-04 | 5.148786569 |
| THBS2 | 1.121715796 | 7.585303389 | 3.442859591 | 9.699E-04 | 1.16E-02 | -0.929974627 |
| GBP5 | 1.121891742 | 7.936674041 | 5.115856257 | 2.559E-06 | 2.77E-04 | 4.560336739 |
| NRCAM | 1.143125463 | 5.926112174 | 6.251078546 | 2.702E-08 | 2.65E-05 | 8.825979952 |
| LILRB2 | 1.148325311 | 8.027143447 | 5.952580308 | 9.185E-08 | 5.06E-05 | 7.677358772 |
| FCGR2A | 1.158269396 | 8.221215027 | 5.258442433 | 1.470E-06 | 1.95E-04 | 5.078603352 |
| IGLV6-57 | 1.16138866 | 6.514667353 | 3.595787575 | 5.942E-04 | 8.29E-03 | -0.48398402 |
| PLEK | 1.170304475 | 9.220882903 | 4.306491474 | 5.238E-05 | 1.77E-03 | 1.75026741 |
| FFAR2 | 1.174408612 | 7.58505761 | 4.320727682 | 4.977E-05 | 1.71E-03 | 1.79747845 |
| COL12A1 | 1.175454124 | 8.177451503 | 4.388460646 | 3.901E-05 | 1.48E-03 | 2.023295801 |
| P2RY13 | 1.196732375 | 7.124776207 | 5.26172934 | 1.451E-06 | 1.95E-04 | 5.090620816 |
| RGS1 | 1.200954161 | 8.074937064 | 4.788436002 | 8.924E-06 | 6.12E-04 | 3.394638157 |
| SLC2A3 | 1.213331007 | 8.695222004 | 4.934460889 | 5.134E-06 | 4.50E-04 | 3.91010719 |
| CXCL11 | 1.215829357 | 8.828676521 | 4.233243755 | 6.799E-05 | 2.01E-03 | 1.508767748 |
| IGFBP5 | 1.218612332 | 9.192114324 | 6.187435742 | 3.511E-08 | 3.09E-05 | 8.579807801 |
| IFI44L | 1.234738224 | 6.794480206 | 5.374669427 | 9.318E-07 | 1.58E-04 | 5.505391381 |
| TAGAP | 1.253745605 | 7.929694063 | 5.788500103 | 1.787E-07 | 6.30E-05 | 7.053070924 |
| MME | 1.257727674 | 7.20166194 | 5.80911672 | 1.644E-07 | 5.91E-05 | 7.131208681 |
| SELL | 1.289190698 | 9.347137811 | 4.743353086 | 1.057E-05 | 6.93E-04 | 3.237014564 |
| IL18RAP | 1.300350402 | 6.99351597 | 6.063945232 | 5.829E-08 | 4.63E-05 | 8.104061946 |
| SERPINE1 | 1.311333849 | 7.93311972 | 5.834295256 | 1.485E-07 | 5.80E-05 | 7.226757634 |
| SERPINB2 | 1.321049783 | 5.845059699 | 3.485554528 | 8.469E-04 | 1.05E-02 | -0.806812811 |
| FAP | 1.327641723 | 6.671660794 | 4.698396907 | 1.250E-05 | 7.52E-04 | 3.080573063 |
| FPR2 | 1.333253295 | 7.018853413 | 4.907128278 | 5.697E-06 | 4.73E-04 | 3.813064561 |
| GLT1D1 | 1.338988517 | 6.323843489 | 5.605612799 | 3.726E-07 | 8.82E-05 | 6.364024564 |
| GPR84 | 1.354694421 | 6.552927739 | 4.52438161 | 2.379E-05 | 1.12E-03 | 2.482247856 |
| IL1B | 1.357659168 | 11.01048103 | 5.477341456 | 6.210E-07 | 1.16E-04 | 5.885445504 |
| CLEC4E | 1.363808892 | 6.505268903 | 6.311131275 | 2.108E-08 | 2.32E-05 | 9.058846247 |
| CYP26B1 | 1.368564918 | 6.201336319 | 5.060095923 | 3.173E-06 | 3.18E-04 | 4.359338906 |
| FPR1 | 1.370569652 | 8.496062384 | 4.946192751 | 4.910E-06 | 4.42E-04 | 3.95183739 |
| CCL2 | 1.378932374 | 9.050998467 | 4.768312374 | 9.625E-06 | 6.45E-04 | 3.324189061 |
| MMP1 | 1.388775787 | 11.40756128 | 4.263697707 | 6.102E-05 | 1.90E-03 | 1.608886552 |
| STC1 | 1.398321207 | 7.880993467 | 7.121717358 | 7.081E-10 | 3.12E-06 | 12.24509577 |
| S100A9 | 1.404901768 | 10.66697504 | 5.28709539 | 1.314E-06 | 1.88E-04 | 5.183466805 |
| WNT5A | 1.40847527 | 9.02847598 | 6.611466873 | 6.050E-09 | 1.18E-05 | 10.23104489 |
| OSM | 1.410857226 | 7.456838543 | 4.555910425 | 2.119E-05 | 1.04E-03 | 2.589780482 |
| TLR2 | 1.417463723 | 7.578634316 | 5.939256222 | 9.697E-08 | 5.15E-05 | 7.626462926 |
| CXCL6 | 1.423147901 | 8.932056051 | 5.23746347 | 1.596E-06 | 2.07E-04 | 5.001974033 |
| CHI3L1 | 1.432089468 | 9.810492077 | 5.031007077 | 3.549E-06 | 3.40E-04 | 4.254871473 |
| GAS1 | 1.439335029 | 6.787171881 | 3.888596984 | 2.248E-04 | 4.36E-03 | 0.405889257 |
| CXCR2 | 1.446052061 | 7.900166826 | 4.611333043 | 1.727E-05 | 9.28E-04 | 2.779754981 |
| MNDA | 1.446815058 | 8.445974231 | 5.264886306 | 1.434E-06 | 1.94E-04 | 5.102166107 |
| KLK10 | 1.4484353 | 8.684758916 | 3.069475226 | 3.038E-03 | 2.53E-02 | -1.960082847 |
| TNFRSF11B | 1.453736583 | 8.181962371 | 7.256681291 | 3.999E-10 | 2.35E-06 | 12.78120181 |
| IRG1 | 1.460701317 | 6.341891183 | 4.869457249 | 6.573E-06 | 5.09E-04 | 3.679732324 |
| S100A8 | 1.461165196 | 10.89581578 | 4.621396263 | 1.664E-05 | 9.03E-04 | 2.814377203 |
| CEMIP | 1.479919462 | 8.552718634 | 4.910152937 | 5.632E-06 | 4.73E-04 | 3.823790991 |
| TNC | 1.495873369 | 8.888548793 | 6.010092771 | 7.265E-08 | 4.92E-05 | 7.897435125 |
| VNN2 | 1.506738113 | 8.027579086 | 5.520300834 | 5.236E-07 | 1.07E-04 | 6.045272987 |
| BCL2A1 | 1.512385427 | 9.294976796 | 5.336038025 | 1.085E-06 | 1.73E-04 | 5.363120462 |
| G0S2 | 1.532155924 | 9.825987076 | 6.055070606 | 6.045E-08 | 4.63E-05 | 8.069974694 |
| CXCL8 | 1.555602379 | 11.05905448 | 5.616623604 | 3.565E-07 | 8.61E-05 | 6.405292155 |
| CXCR1 | 1.58122868 | 6.77198419 | 4.100235816 | 1.086E-04 | 2.66E-03 | 1.07644011 |
| MMP3 | 1.645300871 | 11.54488234 | 5.762413258 | 1.985E-07 | 6.60E-05 | 6.954330922 |
| LILRA3 | 1.650179118 | 6.031881481 | 5.681811911 | 2.746E-07 | 7.56E-05 | 6.650193244 |
| S100A12 | 1.671242935 | 8.268403814 | 4.3914573 | 3.859E-05 | 1.48E-03 | 2.033331644 |
| CXCL5 | 1.704089244 | 9.231971236 | 5.181189507 | 1.987E-06 | 2.35E-04 | 4.797056998 |
| AQP9 | 1.710524746 | 8.660608323 | 4.859553126 | 6.824E-06 | 5.16E-04 | 3.644758837 |
| TREM1 | 1.79006911 | 7.897090973 | 5.557825858 | 4.509E-07 | 9.81E-05 | 6.185259363 |
| TNFAIP6 | 1.791107261 | 8.49468426 | 5.188671532 | 1.930E-06 | 2.31E-04 | 4.824248366 |
| HCAR3 | 1.839134742 | 9.363693406 | 4.833038522 | 7.543E-06 | 5.43E-04 | 3.551298221 |
| SELE | 1.852450332 | 8.013898939 | 5.866196577 | 1.305E-07 | 5.61E-05 | 7.348007196 |
| PROK2 | 1.917484407 | 8.399567237 | 4.923921663 | 5.345E-06 | 4.55E-04 | 3.872658526 |
| INHBA | 1.947690986 | 8.057830977 | 6.348774608 | 1.804E-08 | 2.27E-05 | 9.205091692 |
| IL6 | 1.949260285 | 8.084288689 | 5.891253529 | 1.179E-07 | 5.56E-05 | 7.44338819 |
| PTGS2 | 1.950882958 | 9.093271107 | 6.349066024 | 1.802E-08 | 2.27E-05 | 9.206224663 |
| IL13RA2 | 1.953428363 | 7.447336564 | 7.834009153 | 3.434E-11 | 6.05E-07 | 15.08294854 |
| IL24 | 2.112965045 | 7.19981962 | 6.019073421 | 7.003E-08 | 4.92E-05 | 7.931856244 |
| CSF3 | 2.1403527 | 6.659137799 | 5.695272556 | 2.601E-07 | 7.50E-05 | 6.700884168 |
| PI15 | 2.172099282 | 8.046726859 | 5.690471625 | 2.652E-07 | 7.50E-05 | 6.68279982 |
| IL11 | 2.224933586 | 7.062663623 | 6.752634711 | 3.351E-09 | 8.44E-06 | 10.7858146 |
| TFPI2 | 2.439167468 | 6.51209392 | 6.647987906 | 5.194E-09 | 1.14E-05 | 10.37435753 |

**Supplementary Table 4: Perturbed Genes following virtual knockout of PTGS2 in the colon**

| **Gene** | **Distance** | **Z score** | **Fold Change** | **P value** | **Adjusted P value** |
| --- | --- | --- | --- | --- | --- |
| TYROBP | 9.609149E-09 | 1.815867 | 31.174186 | 2.358820E-08 | 2.953201E-05 |
| HLA-DRA | 9.519365E-09 | 1.811275 | 30.594351 | 3.180184E-08 | 2.953201E-05 |
| CD68 | 9.508421E-09 | 1.810712 | 30.524043 | 3.297544E-08 | 2.953201E-05 |
| IFI30 | 9.504318E-09 | 1.810501 | 30.497706 | 3.342616E-08 | 2.953201E-05 |
| FCER1G | 9.486631E-09 | 1.809591 | 30.384304 | 3.543841E-08 | 2.953201E-05 |
| HLA-DRB1 | 9.181816E-09 | 1.793665 | 28.463118 | 9.550050E-08 | 6.633409E-05 |
| HLA-DPA1 | 9.137142E-09 | 1.791293 | 28.186814 | 1.101524E-07 | 6.633409E-05 |
| CTSB | 9.095664E-09 | 1.789081 | 27.931492 | 1.256874E-07 | 6.633409E-05 |
| AIF1 | 9.059764E-09 | 1.787159 | 27.711437 | 1.408277E-07 | 6.633409E-05 |
| IGSF6 | 9.048492E-09 | 1.786555 | 27.642524 | 1.459350E-07 | 6.633409E-05 |
| CTSS | 8.938122E-09 | 1.780599 | 26.972290 | 2.063932E-07 | 8.599717E-05 |
| C1QC | 8.864132E-09 | 1.776570 | 26.527584 | 2.598018E-07 | 9.992376E-05 |
| HLA-DPB1 | 8.826184E-09 | 1.774493 | 26.300940 | 2.921465E-07 | 1.003477E-04 |
| PSAP | 8.816458E-09 | 1.773959 | 26.243006 | 3.010431E-07 | 1.003477E-04 |
| C1QA | 8.708172E-09 | 1.767982 | 25.602320 | 4.195347E-07 | 1.311046E-04 |
| C1QB | 8.683050E-09 | 1.766586 | 25.454815 | 4.528660E-07 | 1.331959E-04 |
| FTL | 8.599222E-09 | 1.761903 | 24.965690 | 5.835967E-07 | 1.621102E-04 |
| LYZ | 8.581010E-09 | 1.760880 | 24.860058 | 6.164655E-07 | 1.622278E-04 |
| PLA2G7 | 8.488777E-09 | 1.755671 | 24.328514 | 8.122773E-07 | 2.030693E-04 |
| ITGB2 | 8.471084E-09 | 1.754666 | 24.227200 | 8.561460E-07 | 2.038443E-04 |
| NPC2 | 8.441395E-09 | 1.752976 | 24.057680 | 9.349263E-07 | 2.124832E-04 |
| CTSZ | 8.409435E-09 | 1.751151 | 23.875854 | 1.027525E-06 | 2.232736E-04 |
| FGL2 | 8.395149E-09 | 1.750333 | 23.794805 | 1.071713E-06 | 2.232736E-04 |
| RNF130 | 8.358614E-09 | 1.748236 | 23.588145 | 1.193204E-06 | 2.295427E-04 |
| GRN | 8.355823E-09 | 1.748075 | 23.572396 | 1.203010E-06 | 2.295427E-04 |
| HLA-DQA1 | 8.345618E-09 | 1.747488 | 23.514854 | 1.239531E-06 | 2.295427E-04 |
| LST1 | 8.315114E-09 | 1.745728 | 23.343270 | 1.355168E-06 | 2.419943E-04 |
| CXCL16 | 8.195526E-09 | 1.738776 | 22.676653 | 1.916827E-06 | 3.220855E-04 |
| LITAF | 8.192696E-09 | 1.738610 | 22.660994 | 1.932513E-06 | 3.220855E-04 |
| HLA-DMB | 8.159943E-09 | 1.736690 | 22.480167 | 2.123245E-06 | 3.424589E-04 |
| FCGR2A | 8.103179E-09 | 1.733347 | 22.168493 | 2.497387E-06 | 3.902168E-04 |
| CD74 | 8.077194E-09 | 1.731809 | 22.026544 | 2.689058E-06 | 4.074331E-04 |
| MPEG1 | 7.989753E-09 | 1.726604 | 21.552220 | 3.443238E-06 | 5.023560E-04 |
| CYBB | 7.982266E-09 | 1.726156 | 21.511849 | 3.516492E-06 | 5.023560E-04 |
| CTSL | 7.962789E-09 | 1.724989 | 21.406994 | 3.714139E-06 | 5.158527E-04 |
| CPVL | 7.942359E-09 | 1.723762 | 21.297292 | 3.932868E-06 | 5.314686E-04 |
| CST3 | 7.909892E-09 | 1.721807 | 21.123527 | 4.306088E-06 | 5.665905E-04 |
| FTH1 | 7.898236E-09 | 1.721103 | 21.061318 | 4.448177E-06 | 5.702791E-04 |
| CD14 | 7.872599E-09 | 1.719552 | 20.924811 | 4.776676E-06 | 5.970845E-04 |
| MS4A6A | 7.862723E-09 | 1.718953 | 20.872347 | 4.909310E-06 | 5.986964E-04 |
| CLEC7A | 7.831660E-09 | 1.717066 | 20.707750 | 5.349905E-06 | 6.368934E-04 |
| GSTO1 | 7.803520E-09 | 1.715351 | 20.559208 | 5.781502E-06 | 6.616545E-04 |
| NAMPT | 7.800949E-09 | 1.715194 | 20.545662 | 5.822560E-06 | 6.616545E-04 |
| FCGRT | 7.785439E-09 | 1.714246 | 20.464049 | 6.076191E-06 | 6.751323E-04 |
| S100A11 | 7.621716E-09 | 1.704137 | 19.612406 | 9.485133E-06 | 1.015247E-03 |
| CTSD | 7.619445E-09 | 1.703996 | 19.600719 | 9.543325E-06 | 1.015247E-03 |
| CD4 | 7.551477E-09 | 1.699743 | 19.252589 | 1.145150E-05 | 1.187551E-03 |
| TNFSF13B | 7.545426E-09 | 1.699362 | 19.221748 | 1.163800E-05 | 1.187551E-03 |
| HLA-DQB1 | 7.504127E-09 | 1.696760 | 19.011905 | 1.299054E-05 | 1.299054E-03 |
| LIPA | 7.453670E-09 | 1.693563 | 18.757096 | 1.484698E-05 | 1.455587E-03 |
| HLA-DMA | 7.418799E-09 | 1.691343 | 18.582004 | 1.627497E-05 | 1.564901E-03 |
| ASAH1 | 7.403779E-09 | 1.690384 | 18.506838 | 1.692958E-05 | 1.596280E-03 |
| ACP5 | 7.396853E-09 | 1.689941 | 18.472228 | 1.723983E-05 | 1.596280E-03 |
| CSTA | 7.386075E-09 | 1.689252 | 18.418432 | 1.773343E-05 | 1.612130E-03 |
| C5AR1 | 7.319055E-09 | 1.684942 | 18.085701 | 2.111814E-05 | 1.850798E-03 |
| PLSCR1 | 7.312872E-09 | 1.684543 | 18.055154 | 2.145969E-05 | 1.850798E-03 |
| ITM2B | 7.312700E-09 | 1.684532 | 18.054305 | 2.146926E-05 | 1.850798E-03 |
| CSF1R | 7.296795E-09 | 1.683504 | 17.975855 | 2.237248E-05 | 1.895973E-03 |
| SPI1 | 7.234120E-09 | 1.679432 | 17.668379 | 2.629620E-05 | 2.191350E-03 |
| MS4A4A | 7.208971E-09 | 1.677789 | 17.545747 | 2.804774E-05 | 2.298995E-03 |
| HCK | 7.183073E-09 | 1.676093 | 17.419910 | 2.996705E-05 | 2.416697E-03 |
| HBEGF | 7.171940E-09 | 1.675362 | 17.365952 | 3.082997E-05 | 2.446823E-03 |
| TMSB10 | 7.154730E-09 | 1.674230 | 17.282709 | 3.221046E-05 | 2.516442E-03 |
| MS4A7 | 7.137823E-09 | 1.673116 | 17.201126 | 3.362371E-05 | 2.586439E-03 |
| SAT1 | 7.096619E-09 | 1.670390 | 17.003108 | 3.731867E-05 | 2.827172E-03 |
| CREG1 | 7.046464E-09 | 1.667054 | 16.763617 | 4.233734E-05 | 3.159503E-03 |
| PLEK | 7.034640E-09 | 1.666265 | 16.707405 | 4.361045E-05 | 3.206651E-03 |
| CD163 | 6.998196E-09 | 1.663824 | 16.534745 | 4.776673E-05 | 3.461357E-03 |
| CALHM6 | 6.979759E-09 | 1.662585 | 16.447736 | 5.000987E-05 | 3.572134E-03 |
| MRC1 | 6.952264E-09 | 1.660733 | 16.318407 | 5.354134E-05 | 3.770517E-03 |
| SLAMF8 | 6.933819E-09 | 1.659486 | 16.231937 | 5.604135E-05 | 3.888421E-03 |
| AOAH | 6.928584E-09 | 1.659132 | 16.207432 | 5.677095E-05 | 3.888421E-03 |
| FCGR1A | 6.908827E-09 | 1.657792 | 16.115132 | 5.960586E-05 | 4.027423E-03 |
| LGALS9 | 6.862862E-09 | 1.654664 | 15.901417 | 6.672874E-05 | 4.448583E-03 |
| NPL | 6.841696E-09 | 1.653217 | 15.803485 | 7.027295E-05 | 4.623221E-03 |
| C1orf54 | 6.795448E-09 | 1.650042 | 15.590551 | 7.864662E-05 | 5.106924E-03 |
| SDSL | 6.775448E-09 | 1.648663 | 15.498918 | 8.255248E-05 | 5.291826E-03 |
| PTPRE | 6.767558E-09 | 1.648118 | 15.462840 | 8.414334E-05 | 5.296275E-03 |
| GNS | 6.764633E-09 | 1.647916 | 15.449477 | 8.474040E-05 | 5.296275E-03 |
| FCGR3A | 6.731441E-09 | 1.645616 | 15.298238 | 9.180212E-05 | 5.666798E-03 |
| SDS | 6.721762E-09 | 1.644944 | 15.254277 | 9.396375E-05 | 5.669745E-03 |
| FPR3 | 6.721081E-09 | 1.644896 | 15.251184 | 9.411776E-05 | 5.669745E-03 |
| RAB20 | 6.682148E-09 | 1.642183 | 15.075006 | 1.033223E-04 | 6.150139E-03 |
| CCL4 | 6.674355E-09 | 1.641638 | 15.039865 | 1.052640E-04 | 6.192000E-03 |
| C3AR1 | 6.667691E-09 | 1.641172 | 15.009845 | 1.069518E-04 | 6.218127E-03 |
| CCL4L2 | 6.652495E-09 | 1.640107 | 14.941507 | 1.108961E-04 | 6.373340E-03 |
| SIGLEC7 | 6.646163E-09 | 1.639663 | 14.913077 | 1.125799E-04 | 6.396583E-03 |
| CCRL2 | 6.636056E-09 | 1.638953 | 14.867753 | 1.153175E-04 | 6.478514E-03 |
| APOC1 | 6.625450E-09 | 1.638207 | 14.820266 | 1.182578E-04 | 6.569878E-03 |
| PLAUR | 6.615987E-09 | 1.637540 | 14.777963 | 1.209406E-04 | 6.645090E-03 |
| GPR183 | 6.601406E-09 | 1.636511 | 14.712895 | 1.251872E-04 | 6.803654E-03 |
| PLD3 | 6.590074E-09 | 1.635711 | 14.662429 | 1.285840E-04 | 6.913116E-03 |
| PILRA | 6.573082E-09 | 1.634508 | 14.586915 | 1.338407E-04 | 7.112958E-03 |
| RAB7B | 6.568960E-09 | 1.634215 | 14.568624 | 1.351462E-04 | 7.112958E-03 |
| MNDA | 6.558090E-09 | 1.633444 | 14.520448 | 1.386465E-04 | 7.221170E-03 |
| IL18BP | 6.551327E-09 | 1.632963 | 14.490516 | 1.408670E-04 | 7.261185E-03 |
| THBD | 6.533035E-09 | 1.631662 | 14.409712 | 1.470420E-04 | 7.435071E-03 |
| VSIG4 | 6.532535E-09 | 1.631626 | 14.407505 | 1.472144E-04 | 7.435071E-03 |
| RBM47 | 6.493406E-09 | 1.628830 | 14.235425 | 1.613052E-04 | 8.026451E-03 |
| LGMN | 6.491205E-09 | 1.628673 | 14.225777 | 1.621343E-04 | 8.026451E-03 |
| CXCL8 | 6.472536E-09 | 1.627333 | 14.144067 | 1.693303E-04 | 8.300503E-03 |
| ZFYVE16 | 6.457747E-09 | 1.626270 | 14.079505 | 1.752429E-04 | 8.435027E-03 |
| LAIR1 | 6.457241E-09 | 1.626233 | 14.077299 | 1.754486E-04 | 8.435027E-03 |
| LILRB2 | 6.429841E-09 | 1.624257 | 13.958082 | 1.869323E-04 | 8.901538E-03 |
| TYMP | 6.412108E-09 | 1.622974 | 13.881197 | 1.947370E-04 | 9.185708E-03 |
| CD300A | 6.395489E-09 | 1.621769 | 13.809337 | 2.023282E-04 | 9.454589E-03 |
| JAML | 6.380738E-09 | 1.620698 | 13.745708 | 2.092985E-04 | 9.689745E-03 |
| CLEC4A | 6.372259E-09 | 1.620081 | 13.709203 | 2.134062E-04 | 9.789275E-03 |
| STAB1 | 6.351415E-09 | 1.618561 | 13.619662 | 2.238290E-04 | 1.013175E-02 |
| LILRB4 | 6.349277E-09 | 1.618404 | 13.610493 | 2.249249E-04 | 1.013175E-02 |
| IER3 | 6.335276E-09 | 1.617381 | 13.550534 | 2.322254E-04 | 1.036720E-02 |
| TTYH3 | 6.328922E-09 | 1.616915 | 13.523365 | 2.356115E-04 | 1.042529E-02 |
| ADGRE2 | 6.320809E-09 | 1.616321 | 13.488716 | 2.400024E-04 | 1.052642E-02 |
| TREM2 | 6.293799E-09 | 1.614336 | 13.373683 | 2.551798E-04 | 1.109477E-02 |
| DAPK1 | 6.289699E-09 | 1.614035 | 13.356265 | 2.575607E-04 | 1.110176E-02 |
| CSTB | 6.265725E-09 | 1.612266 | 13.254641 | 2.719059E-04 | 1.161991E-02 |
| LAMP1 | 6.255145E-09 | 1.611484 | 13.209917 | 2.784718E-04 | 1.179965E-02 |
| ARL4C | 6.248179E-09 | 1.610968 | 13.180511 | 2.828756E-04 | 1.180211E-02 |
| CHMP1B | 6.247591E-09 | 1.610924 | 13.178028 | 2.832507E-04 | 1.180211E-02 |
| RAB31 | 6.228607E-09 | 1.609516 | 13.098066 | 2.956009E-04 | 1.211809E-02 |
| CAPG | 6.228486E-09 | 1.609507 | 13.097555 | 2.956814E-04 | 1.211809E-02 |
| RNASE6 | 6.223977E-09 | 1.609172 | 13.078598 | 2.986890E-04 | 1.214183E-02 |
| TFRC | 6.201961E-09 | 1.607533 | 12.986239 | 3.137887E-04 | 1.265277E-02 |
| NINJ1 | 6.185220E-09 | 1.606284 | 12.916223 | 3.257464E-04 | 1.293152E-02 |
| ADAM9 | 6.183351E-09 | 1.606144 | 12.908419 | 3.271075E-04 | 1.293152E-02 |
| SELENOP | 6.179469E-09 | 1.605854 | 12.892217 | 3.299514E-04 | 1.293152E-02 |
| ITGAX | 6.177982E-09 | 1.605742 | 12.886013 | 3.310469E-04 | 1.293152E-02 |
| SLCO2B1 | 6.165823E-09 | 1.604832 | 12.835339 | 3.401338E-04 | 1.318348E-02 |
| SIRPA | 6.161407E-09 | 1.604501 | 12.816962 | 3.434910E-04 | 1.321119E-02 |
| ADAP2 | 6.156000E-09 | 1.604095 | 12.794478 | 3.476440E-04 | 1.326886E-02 |
| BLVRB | 6.103144E-09 | 1.600114 | 12.575709 | 3.907931E-04 | 1.480277E-02 |
| HNMT | 6.094289E-09 | 1.599444 | 12.539246 | 3.984931E-04 | 1.498094E-02 |
| PLIN2 | 6.063968E-09 | 1.597144 | 12.414783 | 4.259485E-04 | 1.589360E-02 |
| CXCL2 | 6.058402E-09 | 1.596721 | 12.392003 | 4.311764E-04 | 1.596950E-02 |
| BCL2A1 | 6.026966E-09 | 1.594323 | 12.263734 | 4.618465E-04 | 1.697965E-02 |
| STAT1 | 6.008183E-09 | 1.592886 | 12.187417 | 4.811296E-04 | 1.755948E-02 |
| SH2B3 | 6.004333E-09 | 1.592591 | 12.171799 | 4.851748E-04 | 1.757880E-02 |
| RASSF4 | 5.991902E-09 | 1.591637 | 12.121454 | 4.984503E-04 | 1.781421E-02 |
| SERPINA1 | 5.986292E-09 | 1.591205 | 12.098768 | 5.045516E-04 | 1.781421E-02 |
| SGK1 | 5.984360E-09 | 1.591057 | 12.090958 | 5.066692E-04 | 1.781421E-02 |
| SOD2 | 5.981617E-09 | 1.590846 | 12.079878 | 5.096894E-04 | 1.781421E-02 |
| SLC8A1 | 5.980299E-09 | 1.590744 | 12.074552 | 5.111475E-04 | 1.781421E-02 |
| INSIG1 | 5.978584E-09 | 1.590612 | 12.067628 | 5.130493E-04 | 1.781421E-02 |
| EFHD2 | 5.924751E-09 | 1.586453 | 11.851287 | 5.762150E-04 | 1.986948E-02 |
| CCL3L1 | 5.904051E-09 | 1.584844 | 11.768619 | 6.023764E-04 | 2.062933E-02 |
| EPB41L3 | 5.898659E-09 | 1.584424 | 11.747134 | 6.093699E-04 | 2.072687E-02 |
| CTSC | 5.882853E-09 | 1.583192 | 11.684262 | 6.303098E-04 | 2.129425E-02 |
| CCL3 | 5.867448E-09 | 1.581988 | 11.623147 | 6.513609E-04 | 2.185775E-02 |
| PLXDC2 | 5.855403E-09 | 1.581045 | 11.575478 | 6.682725E-04 | 2.227575E-02 |
| ANXA5 | 5.812749E-09 | 1.577691 | 11.407447 | 7.315029E-04 | 2.419536E-02 |
| TGFBI | 5.810144E-09 | 1.577486 | 11.397225 | 7.355388E-04 | 2.419536E-02 |
| GLUL | 5.798503E-09 | 1.576566 | 11.351599 | 7.538305E-04 | 2.463498E-02 |
| P2RY6 | 5.778467E-09 | 1.574980 | 11.273285 | 7.863044E-04 | 2.552937E-02 |
| SAMHD1 | 5.773313E-09 | 1.574571 | 11.253188 | 7.948639E-04 | 2.555513E-02 |
| CD86 | 5.771844E-09 | 1.574455 | 11.247461 | 7.973200E-04 | 2.555513E-02 |
| MAFB | 5.720573E-09 | 1.570370 | 11.048526 | 8.875776E-04 | 2.826680E-02 |
| GNAQ | 5.701619E-09 | 1.568852 | 10.975435 | 9.232758E-04 | 2.907557E-02 |
| GPNMB | 5.700928E-09 | 1.568796 | 10.972773 | 9.246032E-04 | 2.907557E-02 |
| CXCL3 | 5.686731E-09 | 1.567656 | 10.918191 | 9.522455E-04 | 2.975767E-02 |
| MSR1 | 5.676077E-09 | 1.566799 | 10.877318 | 9.734912E-04 | 3.023265E-02 |
| CD302 | 5.669914E-09 | 1.566302 | 10.853711 | 9.859796E-04 | 3.043147E-02 |
| RAC1 | 5.658918E-09 | 1.565415 | 10.811652 | 1.008633E-03 | 3.088510E-02 |
| C1orf162 | 5.656809E-09 | 1.565245 | 10.803596 | 1.013031E-03 | 3.088510E-02 |
| IL13RA1 | 5.651990E-09 | 1.564856 | 10.785198 | 1.023149E-03 | 3.100453E-02 |
| CSF2RA | 5.648942E-09 | 1.564609 | 10.773568 | 1.029598E-03 | 3.101199E-02 |
| GK | 5.630480E-09 | 1.563114 | 10.703262 | 1.069468E-03 | 3.202001E-02 |
| BRI3 | 5.620833E-09 | 1.562331 | 10.666616 | 1.090865E-03 | 3.246622E-02 |
| CD63 | 5.575867E-09 | 1.558666 | 10.496636 | 1.195921E-03 | 3.538227E-02 |
| NR4A3 | 5.565427E-09 | 1.557812 | 10.457366 | 1.221614E-03 | 3.592981E-02 |
| KYNU | 5.556467E-09 | 1.557077 | 10.423719 | 1.244071E-03 | 3.637635E-02 |
| ABCA1 | 5.538177E-09 | 1.555575 | 10.355209 | 1.291098E-03 | 3.753193E-02 |
| SDC3 | 5.503068E-09 | 1.552678 | 10.224336 | 1.385996E-03 | 4.005769E-02 |
| SIGLEC12 | 5.477517E-09 | 1.550560 | 10.129612 | 1.459061E-03 | 4.192704E-02 |
| EMILIN2 | 5.458337E-09 | 1.548965 | 10.058795 | 1.516224E-03 | 4.332068E-02 |
| LRRC25 | 5.450497E-09 | 1.548311 | 10.029920 | 1.540177E-03 | 4.375502E-02 |
| FYB1 | 5.422939E-09 | 1.546007 | 9.928755 | 1.627165E-03 | 4.589081E-02 |
| ATOX1 | 5.420921E-09 | 1.545838 | 9.921364 | 1.633713E-03 | 4.589081E-02 |
| GPR34 | 5.412895E-09 | 1.545165 | 9.892007 | 1.659982E-03 | 4.636821E-02 |
| MMP12 | 5.388264E-09 | 1.543094 | 9.802187 | 1.743044E-03 | 4.841790E-02 |

**Supplementary Table 5: PTGS2 expression levels and proportions of PTGS2-positive cells across annotated cell populations in the single-cell RNA-seq dataset**

| Cell types | Average PTGS2 expression | PTGS2-positive cells (%) |
| --- | --- | --- |
| Neurons | 0.02 | 1.01 |
| Monocytes | 2.87 | 41.41 |
| Mast cells | 2.59 | 22.51 |
| Endothelial cells | 0.06 | 4.19 |
| Fibroblasts | 0.60 | 20.48 |
| Macrophages | 0.08 | 0.72 |
| Secretory B cells | 0.01 | 0.21 |
| Epithelial cells | 0.02 | 0.34 |
| B cells | 0.00 | 0.24 |
| CD4^+^ T cells | 0.01 | 0.19 |
| T cells | 0.00 | 0.10 |

**Supplementary Table 6: Bioactive compounds of Gegen Qinlian Decoction (GQD) and their corresponding predicted target genes**

| **Target genes** | **Molecule ID**  **(TCMSP)** |
| --- | --- |
| ABCC1 | MOL004328 |
| ABCG2 | MOL000098，MOL000098 |
| ACACA | MOL000098，MOL000098 |
| ACHE | MOL000354，MOL003896，MOL000392，MOL000422，MOL004808，MOL004811，MOL004824，MOL004827，MOL004833，MOL004856，MOL004884，MOL004885，MOL004904，MOL004908，MOL004912，MOL004924，MOL004974，MOL004978，MOL004991，MOL005003，MOL005007，MOL005008，MOL000098，MOL000392，MOL000098，MOL002915 |
| ACP3 | MOL000098，MOL000098 |
| ADH1C | MOL000449 |
| ADIPOQ | MOL004328 |
| ADRA1A | MOL002565，MOL000392，MOL000500，MOL000392，MOL000358，MOL002903，MOL000228，MOL000358，MOL000449 |
| ADRA1B | MOL001484，MOL002565，MOL003896，MOL000422，MOL004815，MOL004829，MOL004833，MOL004835，MOL004857，MOL004891，MOL004908，MOL004945，MOL004959，MOL004966，MOL000497，MOL004974，MOL004978，MOL004991，MOL000500，MOL005003，MOL000358，MOL002903，MOL002904，MOL000228，MOL002915，MOL002928，MOL002937，MOL000358，MOL000449，MOL008206 |
| ADRA1D | MOL001484，MOL002565，MOL003896，MOL004891，MOL004959，MOL004991，MOL002903，MOL002904 |
| ADRA2A | MOL000449 |
| ADRA2C | MOL002903 |
| ADRB1 | MOL003896，MOL000449 |
| ADRB2 | MOL001484，MOL001792，MOL002565，MOL003896，MOL000392，MOL000417，MOL004833，MOL004835，MOL004841，MOL004857，MOL004866，MOL004891，MOL004908，MOL004911，MOL004941，MOL004945，MOL004957，MOL004959，MOL004966，MOL000497，MOL004974，MOL004978，MOL004980，MOL004991，MOL000500，MOL005003，MOL005020，MOL000098，MOL000392，MOL000358，MOL001454，MOL002903，MOL002904，MOL000785，MOL000098，MOL001689，MOL000173，MOL000228，MOL002915，MOL002928，MOL002937，MOL000358，MOL000449，MOL002879，MOL008206 |
| AHR | MOL000422，MOL000098，MOL000098，MOL002714 |
| AHSA1 | MOL000422，MOL000098，MOL000098，MOL000173 |
| AKR1B1 | MOL000354，MOL000098，MOL000098，MOL000449 |
| AKR1C1 | MOL004328 |
| AKR1C3 | MOL000422 |
| AKT1 | MOL000422，MOL004328，MOL000098，MOL000098，MOL000173，MOL002714 |
| ALOX5 | MOL000422，MOL000098，MOL000098，MOL000098 |
| APOB | MOL004328 |
| APOD | MOL002714 |
| AR | MOL000239，MOL000354，MOL003656，MOL003896，MOL000392，MOL000417，MOL000422，MOL004805，MOL004808，MOL004810，MOL004811，MOL004814，MOL004815，MOL004820，MOL004824，MOL004827，MOL004828，MOL004833，MOL004835，MOL004841，MOL004848，MOL004849，MOL004855，MOL004856，MOL004857，MOL004863，MOL004864，MOL004866，MOL004879，MOL004882，MOL004883，MOL004884，MOL004885，MOL004891，MOL004898，MOL004904，MOL004907，MOL004908，MOL004911，MOL004912，MOL004914，MOL004915，MOL004948，MOL004949，MOL004957，MOL004959，MOL004961，MOL004966，MOL000497，MOL004974，MOL004978，MOL004980，MOL004988，MOL004990，MOL004991，MOL000500，MOL005000，MOL005001，MOL005003，MOL005007，MOL005008，MOL005012，MOL005016，MOL005017，MOL005020，MOL000098，MOL000392，MOL002959，MOL001454，MOL002894，MOL002897，MOL002904，MOL000785，MOL000098，MOL001458，MOL002668，MOL001689，MOL000173，MOL002714，MOL002909，MOL002917，MOL002925，MOL002927，MOL002928，MOL002932，MOL002933，MOL002934，MOL000525，MOL000552，MOL001458，MOL002897，MOL008206，MOL012266 |
| BACE1 | MOL004806 |
| BAD | MOL004328 |
| BAX | MOL000422，MOL000098，MOL000358，MOL000098，MOL001689，MOL000173，MOL002714，MOL000358 |
| BCL2 | MOL000422，MOL004328，MOL000497，MOL000098，MOL000358，MOL000098，MOL001689，MOL000173，MOL002714，MOL002928，MOL000358 |
| BCL2L1 | MOL000098，MOL000098 |
| BIRC5 | MOL000098，MOL000098 |
| CA2 | MOL004815，MOL004835，MOL004841，MOL000497，MOL012245，MOL012246，MOL012266 |
| CACNA2D1 | MOL002927 |
| CAMTA2 | MOL001484，MOL000239，MOL002565，MOL000354，MOL003656，MOL003896，MOL000392，MOL000417，MOL000422，MOL004805，MOL004806，MOL004808，MOL004810，MOL004811，MOL004815，MOL004820，MOL004824，MOL004827，MOL004828，MOL004829，MOL004833，MOL004835，MOL004841，MOL004848，MOL004849，MOL004855，MOL004856，MOL004857，MOL004863，MOL004864，MOL004866，MOL004879，MOL004883，MOL004884，MOL004885，MOL004891，MOL004898，MOL004903，MOL004904，MOL004908，MOL004910，MOL004911，MOL004912，MOL004915，MOL004935，MOL004941，MOL004945，MOL004949，MOL004957，MOL004959，MOL004961，MOL004966，MOL000497，MOL004974，MOL004978，MOL004980，MOL004988，MOL004989，MOL004991，MOL004993，MOL000500，MOL005000，MOL005001，MOL005003，MOL005007，MOL005008，MOL005012，MOL005016，MOL005018，MOL005020，MOL000392，MOL002959，MOL001454，MOL002894，MOL002903，MOL002904，MOL000785，MOL001689，MOL000173，MOL000228，MOL002714，MOL002914，MOL002915，MOL002917，MOL002927，MOL002928，MOL002932，MOL002933，MOL002934，MOL002937，MOL000552，MOL008206，MOL012245，MOL012266 |
| CASP3 | MOL000422，MOL004328，MOL000098，MOL000358，MOL000098，MOL001689，MOL000173，MOL002714，MOL002928，MOL000358 |
| CASP8 | MOL000098，MOL000358，MOL000098，MOL001689，MOL000358 |
| CASP9 | MOL000098，MOL000358，MOL000098，MOL000173，MOL000358 |
| CAT | MOL004328 |
| CAV1 | MOL000098，MOL000098 |
| CCL2 | MOL000098，MOL000098，MOL000173 |
| CCNA2 | MOL002311，MOL002565，MOL000354，MOL003656，MOL003896，MOL000392，MOL000417，MOL004808，MOL004810，MOL004811，MOL004814，MOL004815，MOL004820，MOL004824，MOL004828，MOL004833，MOL004835，MOL004841，MOL004848，MOL004856，MOL004857，MOL004863，MOL004864，MOL004866，MOL004882，MOL004883，MOL004884，MOL004885，MOL004891，MOL004898，MOL004904，MOL004907，MOL004908，MOL004912，MOL004913，MOL004915，MOL004949，MOL004957，MOL004959，MOL004966，MOL000497，MOL004974，MOL004978，MOL000500，MOL005000，MOL005001，MOL005003，MOL005007，MOL005008，MOL005012，MOL005016，MOL005017，MOL005020，MOL000392，MOL002959 |
| CCNB1 | MOL000098，MOL000098，MOL000098，MOL002714，MOL002928 |
| CCND1 | MOL000497，MOL000098，MOL000098，MOL000173 |
| CD40LG | MOL000098，MOL000098 |
| CDK1 | MOL000422，MOL000098，MOL000098，MOL002714，MOL002928 |
| CDK2 | MOL000239，MOL002565，MOL000354，MOL003656，MOL003896，MOL000392，MOL000417，MOL004808，MOL004810，MOL004811，MOL004814，MOL004815，MOL004820，MOL004824，MOL004827，MOL004828，MOL004833，MOL004835，MOL004841，MOL004848，MOL004849，MOL004863，MOL004864，MOL004866，MOL004882，MOL004883，MOL004884，MOL004885，MOL004891，MOL004898，MOL004904，MOL004907，MOL004908，MOL004911，MOL004912，MOL004913，MOL004914，MOL004915，MOL004949，MOL004957，MOL004959，MOL004961，MOL004966，MOL000497，MOL004974，MOL004978，MOL004990，MOL004991，MOL000500，MOL005003，MOL005007，MOL005008，MOL005012，MOL005016，MOL005017，MOL005020，MOL000392，MOL002959，MOL000785，MOL001689，MOL000173，MOL002917，MOL002932，MOL002933，MOL000525，MOL008206 |
| CDK4 | MOL000497 |
| CDK7 | MOL002928 |
| CDKN1A | MOL000098，MOL000098，MOL001689，MOL000173 |
| CES1 | MOL004328 |
| CHEK1 | MOL002311，MOL000239，MOL000354，MOL003656，MOL003896，MOL000392，MOL000417，MOL004811，MOL004814，MOL004815，MOL004820，MOL004824，MOL004827，MOL004828，MOL004833，MOL004835，MOL004841，MOL004849，MOL004855，MOL004856，MOL004857，MOL004863，MOL004864，MOL004866，MOL004879，MOL004883，MOL004884，MOL004907，MOL004908，MOL004912，MOL004913，MOL004914，MOL004915，MOL004957，MOL004966，MOL000497，MOL004974，MOL004978，MOL004990，MOL004991，MOL000500，MOL005000，MOL005012，MOL005016，MOL005017，MOL005020，MOL000392，MOL002959，MOL002668，MOL001689，MOL000173，MOL002917，MOL002932，MOL002933，MOL002934，MOL000525，MOL008206 |
| CHEK2 | MOL000098，MOL000098 |
| CHRM1 | MOL001484，MOL002565，MOL003896，MOL000392，MOL000422，MOL004833，MOL004835，MOL004891，MOL004908，MOL004957，MOL000497，MOL004978，MOL000500，MOL005003，MOL000392，MOL000358，MOL002903，MOL000228，MOL000358，MOL000449 |
| CHRM2 | MOL002565，MOL000422，MOL000358，MOL002903，MOL000358，MOL000449 |
| CHRM3 | MOL001484，MOL002565，MOL003896，MOL004891，MOL004978，MOL005003，MOL000358，MOL002903，MOL002904，MOL000228，MOL000358，MOL000449，MOL002879 |
| CHRM4 | MOL002565，MOL000500，MOL000358，MOL002903，MOL000358 |
| CHRM5 | MOL002565，MOL003896，MOL005003，MOL002903 |
| CHRNA2 | MOL000358，MOL000358 |
| CHUK | MOL000098，MOL000098 |
| CLDN4 | MOL000098，MOL000098 |
| COL1A1 | MOL000098，MOL000098，MOL000098 |
| COL3A1 | MOL000098，MOL000098 |
| CTRB1 | MOL000449 |
| CTSD | MOL000098，MOL000098 |
| CXCL10 | MOL000098，MOL000098 |
| CXCL11 | MOL000098，MOL000098 |
| CXCL2 | MOL000098，MOL000098 |
| CXCL8 | MOL000098，MOL000098，MOL000173 |
| CYCS | MOL002714 |
| CYP19A1 | MOL004328，MOL001689 |
| CYP1A1 | MOL000422，MOL000098，MOL000098 |
| CYP1A2 | MOL000422，MOL000098，MOL000098，MOL002928 |
| CYP1B1 | MOL000422，MOL000098，MOL000098，MOL000098 |
| CYP2C9 | MOL002928 |
| CYP3A4 | MOL000422，MOL000098，MOL000098 |
| DCAF5 | MOL000098，MOL000098 |
| DIO1 | MOL000422，MOL000098，MOL000098 |
| DPEP1 | MOL001792，MOL000392，MOL004328，MOL004941，MOL000392，MOL000073 |
| DPP4 | MOL000239，MOL002565，MOL000354，MOL003656，MOL003896，MOL000392，MOL000417，MOL000422，MOL004808，MOL004811，MOL004824，MOL004828，MOL004849，MOL004856，MOL004857，MOL004864，MOL004866，MOL004879，MOL004883，MOL004907，MOL004912，MOL004915，MOL004948，MOL004957，MOL004961，MOL004980，MOL004990，MOL004991，MOL000500，MOL005000，MOL005008，MOL005012，MOL005016，MOL000098，MOL000392，MOL000098，MOL001689，MOL000173，MOL002714，MOL002909，MOL002915，MOL002917，MOL002925，MOL002927，MOL002928，MOL002932，MOL002933，MOL002934，MOL000525，MOL000552，MOL008206，MOL012266 |
| DRD1 | MOL002565，MOL003896，MOL000358，MOL002903，MOL000228，MOL000358 |
| DRD5 | MOL002903 |
| DUOX2 | MOL000098，MOL000098 |
| E2F1 | MOL000098，MOL000098 |
| E2F2 | MOL000098，MOL000098 |
| EGF | MOL000098，MOL000098 |
| EGFR | MOL000098，MOL000098 |
| EGLN1 | MOL002714 |
| EIF6 | MOL000497，MOL000098，MOL000098，MOL000173 |
| ELK1 | MOL000098，MOL000098 |
| ERBB2 | MOL000098，MOL000098 |
| ERBB3 | MOL000098，MOL000098 |
| ESR1 | MOL001792，MOL002311，MOL002565，MOL000354，MOL003656，MOL003896，MOL000392，MOL000417，MOL004328，MOL004805，MOL004806，MOL004808，MOL004810，MOL004811，MOL004814，MOL004815，MOL004820，MOL004824，MOL004827，MOL004828，MOL004829，MOL004833，MOL004835，MOL004838，MOL004841，MOL004848，MOL004849，MOL004855，MOL004856，MOL004857，MOL004863，MOL004864，MOL004879，MOL004882，MOL004883，MOL004884，MOL004885，MOL004891，MOL004898，MOL004904，MOL004907，MOL004908，MOL004910，MOL004911，MOL004912，MOL004913，MOL004914，MOL004915，MOL004935，MOL004941，MOL004945，MOL004948，MOL004949，MOL004957，MOL004959，MOL004961，MOL004966，MOL000497，MOL004974，MOL004978，MOL004980，MOL004988，MOL004989，MOL004990，MOL004991，MOL004993，MOL000500，MOL005000，MOL005001，MOL005003，MOL005007，MOL005008，MOL005012，MOL005016，MOL005017，MOL005018，MOL005020，MOL000392，MOL002959，MOL001454，MOL002894，MOL002897，MOL000785，MOL001458，MOL002668，MOL000173，MOL000228，MOL002933，MOL002934，MOL000073，MOL001458，MOL002897 |
| ESR2 | MOL000239，MOL002565，MOL000354，MOL003656，MOL003896，MOL000392，MOL000417，MOL004805，MOL004806，MOL004808，MOL004810，MOL004811，MOL004814，MOL004815，MOL004820，MOL004824，MOL004833，MOL004835，MOL004841，MOL004848，MOL004849，MOL004856，MOL004857，MOL004864，MOL004879，MOL004882，MOL004884，MOL004885，MOL004891，MOL004907，MOL004908，MOL004911，MOL004912，MOL004913，MOL004915，MOL004945，MOL004957，MOL004959，MOL004961，MOL004966，MOL000497，MOL004974，MOL004978，MOL004988，MOL004990，MOL000500，MOL005000，MOL005003，MOL005007，MOL005008，MOL005012，MOL005016，MOL005018，MOL005020，MOL000392，MOL002959，MOL000785，MOL002917，MOL002932，MOL002934，MOL000552，MOL008206，MOL012266 |
| F3 | MOL000098，MOL000098 |
| F7 | MOL000354，MOL000422，MOL004808，MOL004824，MOL004827，MOL004828，MOL004829，MOL004849，MOL004857，MOL004866，MOL004885，MOL004903，MOL004904，MOL004966，MOL004974，MOL004989，MOL004993，MOL005007，MOL005008，MOL000098，MOL002904，MOL000785，MOL000098，MOL002915，MOL002927，MOL002934，MOL000552，MOL012266 |
| FABP5 | MOL002714 |
| FASLG | MOL001689 |
| FASN | MOL004328，MOL001689 |
| FOS | MOL000098，MOL000098，MOL002714 |
| FOSL1 | MOL002714 |
| FOSL2 | MOL000497，MOL002714 |
| FSD2 | MOL000173 |
| GABRA1 | MOL000354，MOL003896，MOL000422，MOL004941，MOL004991，MOL000098，MOL000358，MOL000622，MOL000098，MOL000173，MOL000228，MOL000358，MOL000449，MOL008206 |
| GJA1 | MOL000098，MOL000098 |
| GOT1 | MOL004328 |
| GRIA2 | MOL000354，MOL000622 |
| GSK3B | MOL002311，MOL000354，MOL003656，MOL003896，MOL000392，MOL000417，MOL004805，MOL004808，MOL004810，MOL004811，MOL004814，MOL004815，MOL004820，MOL004824，MOL004827，MOL004828，MOL004833，MOL004835，MOL004841，MOL004848，MOL004849，MOL004856，MOL004857，MOL004863，MOL004864，MOL004882，MOL004884，MOL004885，MOL004891，MOL004898，MOL004907，MOL004908，MOL004911，MOL004912，MOL004913，MOL004914，MOL004915，MOL004948，MOL004949，MOL004957，MOL004959，MOL004961，MOL004966，MOL000497，MOL004974，MOL004978，MOL004990，MOL004991，MOL000500，MOL005000，MOL005003，MOL005007，MOL005008，MOL005012，MOL005016，MOL005017，MOL000392，MOL002959，MOL000173，MOL002933，MOL002934，MOL008206 |
| GSR | MOL004328 |
| GSTM1 | MOL000422，MOL000098，MOL000098 |
| GSTM2 | MOL000422，MOL000098，MOL000098 |
| GSTP1 | MOL000422，MOL004328，MOL000098，MOL000098 |
| HAS2 | MOL000422，MOL000098，MOL000098 |
| HIF1A | MOL000098，MOL000098，MOL002714 |
| HK2 | MOL000098，MOL000098 |
| HMGCR | MOL004328 |
| HMOX1 | MOL000422，MOL000098，MOL000098 |
| HSD3B1 | MOL000392，MOL000392 |
| HSD3B2 | MOL000392，MOL000392 |
| HSF1 | MOL000098，MOL000098 |
| HSP90AA1 | MOL001484，MOL001792，MOL000239，MOL002565，MOL000354，MOL003656，MOL003896，MOL000392，MOL000417，MOL000422，MOL004328，MOL004808，MOL004810，MOL004811，MOL004814，MOL004827，MOL004828，MOL004829，MOL004835，MOL004838，MOL004841，MOL004848，MOL004849，MOL004856，MOL004857，MOL004863，MOL004864，MOL004866，MOL004882，MOL004883，MOL004885，MOL004898，MOL004907，MOL004910，MOL004911，MOL004913，MOL004914，MOL004915，MOL004935，MOL004941，MOL004945，MOL004949，MOL004959，MOL004961，MOL004966，MOL000497，MOL004974，MOL004980，MOL004989，MOL004990，MOL004991，MOL004993，MOL000500，MOL005000，MOL005001，MOL005003，MOL005007，MOL005008，MOL005016，MOL005017，MOL005020，MOL000098，MOL000392，MOL000358，MOL002959，MOL001454，MOL002903，MOL002904，MOL000785，MOL000098，MOL001689，MOL000173，MOL000228，MOL002714，MOL002909，MOL002910，MOL002913，MOL002914，MOL002915，MOL002917，MOL002925，MOL002927，MOL002928，MOL002932，MOL002933，MOL002934，MOL002937，MOL000358，MOL000525，MOL000552，MOL000073，MOL008206，MOL012245，MOL012246，MOL012266 |
| HSPA5 | MOL000098，MOL000098 |
| HSPB1 | MOL000098，MOL000098 |
| HTR3A | MOL001484，MOL004891，MOL002903 |
| ICAM1 | MOL000422，MOL000098，MOL000098 |
| IFNG | MOL000098，MOL000098，MOL000098 |
| IGF2 | MOL000098，MOL000098，MOL002714 |
| IGFBP3 | MOL000098，MOL000098 |
| IGHG1 | MOL001484，MOL003896，MOL004828，MOL004829，MOL004848，MOL004908，MOL004957，MOL002915，MOL002927，MOL000449 |
| IKBKB | MOL000422 |
| IL10 | MOL000098，MOL000098 |
| IL1A | MOL000098，MOL000098，MOL000098 |
| IL1B | MOL000098，MOL000098 |
| IL2RA | MOL000098，MOL000098，MOL000098 |
| IL4 | MOL000392，MOL000392 |
| IL6 | MOL000098，MOL000098，MOL000173，MOL002928 |
| INSR | MOL000422，MOL000098，MOL000098 |
| IRF1 | MOL000098，MOL000098 |
| KCNH2 | MOL004805，MOL004806，MOL004811，MOL004849，MOL004855，MOL004864，MOL004879，MOL004891，MOL004959，MOL004966，MOL004974，MOL004978，MOL005003，MOL005007，MOL000098，MOL000358，MOL001454，MOL002894，MOL002897，MOL002903，MOL002904，MOL000785，MOL000098，MOL001458，MOL002927，MOL002934，MOL000358，MOL000552，MOL001458，MOL002897，MOL012266 |
| KDR | MOL002311，MOL004808，MOL004824，MOL004828，MOL004848，MOL004849，MOL004855，MOL004857，MOL004879，MOL004883，MOL004903，MOL004904，MOL004935，MOL004959，MOL004966，MOL005001，MOL005007，MOL005017，MOL000173，MOL002927，MOL000552，MOL012266 |
| LDLR | MOL004328 |
| LTA4H | MOL003896，MOL004835，MOL000449 |
| MAOA | MOL000449 |
| MAOB | MOL001792，MOL000354，MOL003896，MOL000392，MOL004835，MOL004941，MOL004957，MOL000098，MOL000392，MOL000098，MOL000228，MOL000449 |
| MAP2 | MOL000358，MOL000358 |
| MAPK1 | MOL004328，MOL000497，MOL000098，MOL000098 |
| MAPK10 | MOL002565 |
| MAPK14 | MOL002311，MOL000354，MOL003656，MOL003896，MOL000392，MOL000417，MOL004805，MOL004810，MOL004811，MOL004814，MOL004815，MOL004820，MOL004824，MOL004828，MOL004833，MOL004835，MOL004841，MOL004848，MOL004849，MOL004863，MOL004864，MOL004883，MOL004891，MOL004898，MOL004907，MOL004908，MOL004911，MOL004912，MOL004913，MOL004914，MOL004915，MOL004957，MOL004959，MOL004961，MOL004966，MOL000497，MOL004974，MOL004978，MOL004990，MOL004991，MOL000500，MOL005000，MOL005003，MOL005012，MOL005016，MOL005017，MOL005020，MOL000392，MOL002959，MOL000173，MOL002933，MOL008206 |
| MAPK3 | MOL004328 |
| MAPK8 | MOL000422 |
| MCL1 | MOL000173 |
| MMP1 | MOL000422，MOL000098，MOL000098，MOL000173 |
| MMP2 | MOL000098，MOL000098 |
| MMP3 | MOL000098，MOL000098 |
| MMP9 | MOL000098，MOL000098，MOL002714 |
| MPO | MOL000098，MOL000098，MOL000098，MOL002714 |
| MTTP | MOL004328 |
| MYC | MOL000098，MOL000098 |
| NCF1 | MOL000354，MOL000098，MOL000098 |
| NCOA1 | MOL000354，MOL003896，MOL004820，MOL004829，MOL004833，MOL004835，MOL004849，MOL004885，MOL004891，MOL004908，MOL004959，MOL004966，MOL004974，MOL004978，MOL004993，MOL005007，MOL002959，MOL001689，MOL002714，MOL002927，MOL002928，MOL002932，MOL002937，MOL000552，MOL000449，MOL008206，MOL012266 |
| NCOA2 | MOL000239，MOL000354，MOL000359，MOL003656，MOL003896，MOL000417，MOL000422，MOL004808，MOL004811，MOL004815，MOL004820，MOL004848，MOL004849，MOL004855，MOL004856，MOL004857，MOL004863，MOL004864，MOL004879，MOL004883，MOL004898，MOL004908，MOL004911，MOL004949，MOL004959，MOL004961，MOL004966，MOL000497，MOL004974，MOL004978，MOL004980，MOL004985，MOL004988，MOL004991，MOL004996，MOL005000，MOL005001，MOL005003，MOL005007，MOL005016，MOL005018，MOL005020，MOL000098，MOL000358，MOL001454，MOL002894，MOL002897，MOL002904，MOL002907，MOL000785，MOL000098，MOL001689，MOL002714，MOL002909，MOL002914，MOL002915，MOL002917，MOL002927，MOL002928，MOL002933，MOL002934，MOL000358，MOL000359，MOL000552，MOL000449，MOL002897，MOL010415，MOL012266 |
| NFATC1 | MOL002714 |
| NFE2L2 | MOL000098，MOL000098 |
| NFKBIA | MOL000098，MOL000098 |
| NOS2 | MOL002311，MOL000239，MOL002565，MOL000354，MOL003656，MOL003896，MOL000392，MOL000417，MOL000422，MOL004805，MOL004806，MOL004808，MOL004810，MOL004811，MOL004814，MOL004815，MOL004820，MOL004824，MOL004827，MOL004828，MOL004833，MOL004835，MOL004838，MOL004841，MOL004848，MOL004849，MOL004855，MOL004856，MOL004857，MOL004863，MOL004864，MOL004879，MOL004883，MOL004884，MOL004885，MOL004891，MOL004904，MOL004907，MOL004908，MOL004910，MOL004911，MOL004912，MOL004915，MOL004945，MOL004948，MOL004949，MOL004957，MOL004959，MOL004961，MOL004966，MOL000497，MOL004974，MOL004978，MOL004989，MOL004990，MOL004991，MOL000500，MOL005000，MOL005003，MOL005007，MOL005008，MOL005012，MOL005016，MOL005018，MOL005020，MOL000392，MOL002959，MOL001454，MOL002894，MOL002897，MOL002904，MOL000785，MOL001458，MOL002668，MOL001689，MOL000173，MOL002909，MOL002915，MOL002917，MOL002927，MOL002928，MOL002932，MOL002933，MOL002934，MOL000525，MOL000552，MOL001458，MOL002897，MOL008206，MOL012266 |
| NOS3 | MOL000098，MOL000098 |
| NOX5 | MOL002714 |
| NPEPPS | MOL000098，MOL000098 |
| NQO1 | MOL000098，MOL000098 |
| NR1I2 | MOL000422，MOL000098，MOL000098，MOL000098 |
| NR1I3 | MOL000422，MOL000098，MOL000098 |
| NR3C2 | MOL000359，MOL002907，MOL000359，MOL000449 |
| ODC1 | MOL000098，MOL000098 |
| OLR1 | MOL000354 |
| OPRD1 | MOL002565，MOL004891，MOL002903 |
| OPRM1 | MOL001484，MOL002565，MOL003896，MOL004891，MOL000358，MOL002903，MOL000358 |
| PARP1 | MOL000098，MOL000098 |
| PCOLCE | MOL000098，MOL000098 |
| PDE10A | MOL001454，MOL002897，MOL002903，MOL002897 |
| PGR | MOL000211，MOL000359，MOL000422，MOL000358，MOL000358，MOL000359，MOL000449 |
| PKIA | MOL001792，MOL003896，MOL000392，MOL004835，MOL004941，MOL004957，MOL000500，MOL000392，MOL000228，MOL002928 |
| PLAT | MOL000098，MOL000098，MOL000098 |
| PLAU | MOL000098，MOL000098，MOL000449 |
| PLB1 | MOL004328 |
| PON1 | MOL000098，MOL000358，MOL000098，MOL000358 |
| POR | MOL000098，MOL000098 |
| PPARA | MOL004328，MOL000098，MOL000098 |
| PPARD | MOL000354，MOL005007，MOL000098，MOL000098，MOL000552 |
| PPARG | MOL002311，MOL000354，MOL003656，MOL003896，MOL000392，MOL000392，MOL000417，MOL000422，MOL000422，MOL004328，MOL004805，MOL004808，MOL004810，MOL004811，MOL004815，MOL004820，MOL004824，MOL004827，MOL004828，MOL004833，MOL004835，MOL004841，MOL004848，MOL004849，MOL004855，MOL004856，MOL004857，MOL004863，MOL004864，MOL004866，MOL004879，MOL004883，MOL004884，MOL004885，MOL004891，MOL004898，MOL004904，MOL004907，MOL004908，MOL004911，MOL004912，MOL004913，MOL004914，MOL004915，MOL004949，MOL004957，MOL004959，MOL004961，MOL004966，MOL000497，MOL004974，MOL004978，MOL004980，MOL004990，MOL004991，MOL000500，MOL005000，MOL005003，MOL005007，MOL005012，MOL005016，MOL005017，MOL005020，MOL000098，MOL000098，MOL000392，MOL000392，MOL002959，MOL000098，MOL000098，MOL000173，MOL002933，MOL002934，MOL000525，MOL008206 |
| PPP3CA | MOL000422 |
| PRKCA | MOL000098，MOL000358，MOL000098，MOL000358 |
| PRKCB | MOL000098，MOL000098 |
| PRKCD | MOL000173 |
| PRSS1 | MOL001484，MOL000239，MOL002565，MOL000354，MOL003656，MOL003896，MOL000392，MOL000417，MOL000422，MOL004808，MOL004810，MOL004811，MOL004820，MOL004824，MOL004827，MOL004828，MOL004833，MOL004849，MOL004855，MOL004856，MOL004857，MOL004863，MOL004864，MOL004866，MOL004879，MOL004883，MOL004884，MOL004885，MOL004891，MOL004904，MOL004907，MOL004908，MOL004911，MOL004912，MOL004915，MOL004949，MOL004957，MOL004959，MOL004961，MOL004966，MOL004974，MOL004978，MOL004980，MOL004991，MOL000500，MOL005000，MOL005001，MOL005003，MOL005007，MOL005008，MOL005012，MOL005016，MOL005020，MOL000098，MOL000392，MOL002959，MOL001454，MOL002894，MOL002897，MOL002904，MOL000785，MOL000098，MOL001458，MOL001689，MOL000173，MOL002714，MOL002909，MOL002915，MOL002917，MOL002927，MOL002928，MOL002932，MOL002933，MOL002934，MOL000552，MOL001458，MOL002897，MOL008206，MOL012266 |
| PSMD3 | MOL000422，MOL000098，MOL000098 |
| PTGER3 | MOL000098，MOL000098，MOL000173 |
| PTGS1 | MOL001484，MOL001792，MOL000239，MOL002565，MOL000354，MOL003896，MOL000392，MOL000417，MOL000422，MOL004328，MOL004810，MOL004815，MOL004820，MOL004828，MOL004829，MOL004835，MOL004841，MOL004885，MOL004891，MOL004907，MOL004910，MOL004911，MOL004912，MOL004941，MOL004945，MOL004957，MOL004959，MOL004961，MOL004966，MOL000497，MOL004974，MOL004978，MOL004980，MOL004990，MOL004991，MOL000500，MOL005003，MOL005007，MOL005016，MOL000098，MOL000392，MOL000358，MOL002959，MOL001454，MOL002894，MOL002903，MOL002904，MOL000785，MOL000098，MOL001458，MOL002668，MOL001689，MOL000173，MOL000228，MOL002714，MOL002910，MOL002913，MOL002914，MOL002915，MOL002917，MOL002925，MOL002927，MOL002928，MOL002932，MOL002933，MOL002937，MOL000358，MOL000525，MOL000552，MOL000073，MOL000449，MOL001458，MOL008206，MOL012245，MOL012246，MOL012266 |
| PTGS2 | MOL001484，MOL001792，MOL002311，MOL000239，MOL002565，MOL000354，MOL003656，MOL003896，MOL000392，MOL000417，MOL000422，MOL004328，MOL004805，MOL004806，MOL004808，MOL004810，MOL004811，MOL004814，MOL004815，MOL004820，MOL004824，MOL004827，MOL004828，MOL004829，MOL004833，MOL004835，MOL004838，MOL004841，MOL004848，MOL004849，MOL004855，MOL004856，MOL004857，MOL004863，MOL004864，MOL004866，MOL004879，MOL004883，MOL004884，MOL004885，MOL004891，MOL004898，MOL004903，MOL004904，MOL004907，MOL004908，MOL004910，MOL004911，MOL004912，MOL004915，MOL004924，MOL004935，MOL004941，MOL004945，MOL004948，MOL004949，MOL004957，MOL004959，MOL004961，MOL004966，MOL000497，MOL004974，MOL004978，MOL004980，MOL004988，MOL004989，MOL004990，MOL004991，MOL004993，MOL000500，MOL005000，MOL005001，MOL005003，MOL005007，MOL005008，MOL005012，MOL005016，MOL005017，MOL005018，MOL005020，MOL000098，MOL000392，MOL000358，MOL002959，MOL001454，MOL002894，MOL002897，MOL002903，MOL002904，MOL000785，MOL000098，MOL001458，MOL002668，MOL001689，MOL000173，MOL000228，MOL002714，MOL002909，MOL002910，MOL002913，MOL002914，MOL002915，MOL002917，MOL002925，MOL002927，MOL002928，MOL002932，MOL002933，MOL002934，MOL002937，MOL000358，MOL000525，MOL000552，MOL000073，MOL000449，MOL001458，MOL002897，MOL008206，MOL012245，MOL012246，MOL012266 |
| PYGM | MOL000354，MOL002909，MOL002914，MOL002933，MOL002934 |
| RAF1 | MOL000098，MOL000098 |
| RASA1 | MOL000098，MOL000098 |
| RASSF1 | MOL000098，MOL000098 |
| RB1 | MOL000497，MOL000098，MOL000098 |
| RELA | MOL000354，MOL000422，MOL004328，MOL000497，MOL000098，MOL000098，MOL001689，MOL000173，MOL002714 |
| RUNX1T1 | MOL000098，MOL000098 |
| RUNX2 | MOL000098，MOL000098 |
| RXRA | MOL001484，MOL001792，MOL002565，MOL003896，MOL000392，MOL000417，MOL004811，MOL004815，MOL004820，MOL004828，MOL004829，MOL004833，MOL004838，MOL004891，MOL004908，MOL004911，MOL004912，MOL004941，MOL004957，MOL004959，MOL004974，MOL004978，MOL004991，MOL000500，MOL005003，MOL005016，MOL000098，MOL000392，MOL002959，MOL001454，MOL002894，MOL002897，MOL002903，MOL002904，MOL000785，MOL000098，MOL000173，MOL000228，MOL002915，MOL002928，MOL002937，MOL000449，MOL002897，MOL008206，MOL012266 |
| RXRB | MOL004891，MOL004908，MOL004978，MOL005003 |
| SCN5A | MOL001484，MOL000239，MOL002565，MOL003656，MOL003896，MOL004806，MOL004810，MOL004811，MOL004815，MOL004820，MOL004827，MOL004828，MOL004829，MOL004833，MOL004835，MOL004856，MOL004866，MOL004885，MOL004891，MOL004908，MOL004910，MOL004911，MOL004912，MOL004915，MOL004945，MOL004957，MOL004959，MOL004961，MOL004966，MOL000497，MOL004974，MOL004978，MOL004980，MOL004989，MOL004991，MOL004993，MOL000500，MOL005003，MOL005007，MOL005012，MOL005016，MOL005020，MOL000098，MOL000358，MOL001454，MOL002894，MOL002903，MOL002904，MOL000785，MOL000098，MOL001458，MOL000173，MOL000228，MOL002915，MOL002917，MOL002927，MOL002928，MOL002932，MOL002934，MOL002937，MOL000358，MOL000552，MOL000449，MOL001458，MOL001490，MOL002879，MOL008206，MOL012266 |
| SELE | MOL000422，MOL000098，MOL000098 |
| SERPINE1 | MOL000098，MOL000098，MOL000098 |
| SLC2A4 | MOL000422，MOL000098，MOL000098 |
| SLC6A2 | MOL000422，MOL002903，MOL000449 |
| SLC6A3 | MOL002565，MOL003896，MOL000392，MOL004835，MOL004957，MOL000497，MOL004978，MOL000500，MOL000392，MOL002903，MOL000228，MOL000449 |
| SLC6A4 | MOL001792，MOL002565，MOL003896，MOL000392，MOL004835，MOL004941，MOL004957，MOL000500，MOL000392，MOL000358，MOL002903，MOL000228，MOL000358 |
| SLPI | MOL000422 |
| SOAT1 | MOL004328 |
| SOAT2 | MOL004328 |
| SOD1 | MOL004328，MOL004903，MOL000098，MOL000098 |
| SPP1 | MOL000098，MOL000098 |
| SREBF1 | MOL004328 |
| STAT1 | MOL000422，MOL000098，MOL000098 |
| STAT3 | MOL000497 |
| SULT1E1 | MOL000098，MOL000098，MOL000098 |
| Symbol | MolId |
| TDRD7 | MOL002714 |
| TEP1 | MOL000173 |
| THBD | MOL000098，MOL000098，MOL000098 |
| TNF | MOL000422，MOL000098，MOL000098，MOL000173 |
| TOP1 | MOL000098，MOL000098 |
| TOP2A | MOL000098，MOL000098 |
| TP53 | MOL000098，MOL000098，MOL001689，MOL000173，MOL002714 |
| UGT1A1 | MOL004328 |
| VCAM1 | MOL000422，MOL000098，MOL000098 |
